# Reduced vacuolar ATPase protects mice from Friend virus infection – an unintended but instructive effect in *Hif-2a^fl^* mice

**DOI:** 10.1242/jcs.261893

**Published:** 2024-06-28

**Authors:** Timm Schreiber, Nora Koll, Claudia Padberg, Buena de los Reyes, Theresa Quinting, Anna Malyshkina, Eric Metzen, Kathrin Sutter, Joachim Fandrey, Sandra Winning

**Affiliations:** ^1^ Institute of Physiology, University of Duisburg-Essen, 45147 Essen, Germany; ^2^ Institute of Physiology, Pathophysiology and Toxicology and Center for Biomedical Education and Research (ZBAF), University of Witten/Herdecke, 58455 Witten, Germany; ^3^ Institute for Virology, University Hospital Essen, University of Duisburg-Essen, 45147 Essen, Germany; ^4^ Institute for Research on HIV and AIDS-associated Diseases, University Hospital Essen, University of Duisburg-Essen, 45147 Essen, Germany

**Keywords:** Conditional knockout mice, Hypoxia-inducible factors, Retrovirus infection, Vacuolar ATPase

## Abstract

During acute viral infections, innate immune cells invade inflamed tissues and face hypoxic areas. Hypoxia-inducible factors (HIFs) adapt cellular responses towards these conditions. We wanted to investigate the effects of a loss of HIF-2α in macrophages during acute Friend murine leukemia retrovirus (FV) infection in C57BL/6 mice using a Cre/loxP system. Remarkably, mice with floxed *Hif-2a* (*Hif-2a^fl^*; *Hif-2a* is also known as *Epas1*) did not show any signs of FV infection independent of Cre activity. This prevented a detailed analysis of the role of macrophage HIF-2α for FV infection but allowed us to study a model of unexpected FV resistance. *Hif-2a^fl^* mice showed a significant decrease in the expression of the *Atp6v1e2* gene encoding for the E2 subunit of the vacuolar H^+^-ATPase, which resulted in a decreased acidification of lysosomes and limited virus entry into the cell. These findings highlight that the insertion of loxP sites is not always without functional consequences and has established a phenotype in the floxed *Hif-2a* mouse, which is not only unexpected, but unwanted and is of relevance for the use of this mouse strain in (at least virus) experiments.

## Teaser

**Summary:** Insertion of loxP sites around exon2 of the *Hif-2a* gene unintendedly reduced the expression of the *Atp4v1e2* gene located 147 kbp away. This revealed a prominent role of the vacuolar ATPase for Friend virus spread.

## INTRODUCTION

Immune cells infiltrate areas of acute infection, which are often characterized by a lack of oxygen (hypoxia). Oxygen is necessary for a plethora of biological processes. Cells of aerobic organisms therefore have to adapt to hypoxia. They do so by activation of hypoxia-inducible factors (HIFs). The oxygen-dependent α-subunit [HIF-1α, HIF-2α (also known as EPAS1) or the less-studied HIF-3α] of HIF undergoes immediate intracellular degradation whenever oxygen is present (Jaakkola et al., 2001; Ivan et al., 2001; Epstein et al., 2001). This laborious biological regulation of constant production for subsequent degradation enables an instantaneous accumulation of the α-subunit when hypoxia halts degradation. The HIF α-subunit then translocates into the nucleus and dimerizes with the β-subunit (ARNT) to function as a transcription factor (this complex is then called HIF-1 or HIF-2). HIFs induce genes that improve oxygen and energy delivery under hypoxic conditions. A proper response to hypoxia is thus indispensable for immune cells (reviewed, for example, in Taylor et al., 2016 and Winning and Fandrey, 2022). Recently, we investigated the role of HIF-1α in macrophages in a murine model of acute Friend retrovirus (FV) infection (Schreiber et al., 2017). FV is a γ-retroviral complex that causes lethal erythroleukemia and splenomegaly in susceptible mice and which has two components, the Friend murine leukemia virus (F-MuLV), a replication-competent simple γ-retrovirus, and the spleen focus-forming virus (SFFV), which is the replication-deficient but predominant pathogenic component of the viral complex. FV is able to infect any dividing murine cell (except from hepatocytes, which lack the mCAT-1 receptor that is crucial for viral entry; Closs et al., 1993) and it also targets rapidly dividing immune cells. However, the main objects of viral desire are erythroid precursor cells as well as myeloid cells and B cells (Windmann et al., 2019). SFFV encodes a defective env protein (gp55) showing oncogenic properties. The interaction of gp55 with (among other proteins) the erythropoietin receptor potently drives erythroid precursor cell proliferation leading to splenomegaly (Li et al., 1987). Mouse strains that are resistant to FV-induced diseases (e.g. C57BL/6) are able to recover from FV infection due to a strong immune response in the acute phase of the infection (Dittmer et al., 2019). A massive erythrocyte precursor cell proliferation is also characteristic for acute FV infection in immunocompetent mice, which we recently found was absent when myeloid cells lacked HIF-1α (Schreiber et al., 2017). This was due to the impaired invasion of HIF-1α-deficient macrophages into the spleen. As HIF-1 and HIF-2 have distinct roles in other inflammatory models (Kerber et al., 2020), we wanted to analyze the role of myeloid HIF-2α during FV infection of C57BL/6 mice. Unexpectedly, we observed that mice only carrying loxP sites around exon 2 of the *Hif-2a* gene were already protected from an acute FV infection, regardless of whether they expressed the Cre recombinase or not.

Susceptibility of mice to FV-induced erythroleukemia is coordinated by the non-immunological *Fv1*, *Fv2* (also known as *Mst1r*), *Fv3*, *Fv4*, *Fv5* and *Fv6* genes as well as by the immunological genes *Rfv1*, *Rfv2* and *Rfv3* (also known as *Apobec3*). For example, elevated *Fv1* levels reduce the level of susceptibility to infection by 100-fold (Pincus et al., 1971a,b). Some of the above named genes alter the immune response, whereas others influence the occurrence of anemia or polycythemia in infected animals (Plata and Lilly, 1979; Odaka and Yamamoto, 1966; Kumar et al., 1978a,b; Kai et al., 1976; Taylor et al., 2001; Shibuya and Mak, 1982a,b; Hasenkrug and Chesebro, 1997; Chesebro and Wehrly, 1979). However, none of these susceptibility or resistance genes provides a full resistance to FV infection.

Oxygen tension can exert a significant effect on viral propagation *in vitro* and possibly *in vivo*. In general, hypoxia restricts the replication of viruses that naturally infect tissues exposed to ambient oxygen (for example simian virus 40 and adenovirus) (Riedinger et al., 1999; Shen and Hermiston, 2005) and induces the propagation of viruses that naturally target tissues exposed to low oxygen concentrations [such as vesicular stomatitis virus (VSV), herpesviruses, human immunodeficiency virus (HIV) and parvovirus B19] (Aghi et al., 2008; Connor et al., 2004; Haque et al., 2003; Pillet et al., 2004; Polonis et al., 1991).

To enter the host cell, viruses need to attach to host cell receptors which are either surface receptors or transmembrane transporters (Jobbagy et al., 1999). Binding of MuLV to host cell receptors triggers conformational changes in the envelope proteins that lead to the entry of the virus core into the cell cytoplasm. This occurs either by fusion with the cell membrane or by transition through the endosome (Knoper et al., 2009; Dimitrov, 2004; Maetzig et al., 2011). Ecotropic MuLVs are considered to be pH-dependent viruses and therefore to use the latter pathway (Katen et al., 2001). This pathway involves endosome acidification and occurs at either a mildly acidic pH (6.5–6) in the early endosome or a low pH (5.5–4) in the late endosome and/or lysosome. The acidification of endosomes and lysosomes in eukaryotic cells involves the function of vacuolar H^+^-ATPases (V-ATPases), which are ATP-dependent proton pumps (Nishi and Forgac, 2002; Forgac, 2007). V-ATPases are large multi-subunit complexes composed of a peripheral complex V_1_ (containing eight subunits, A–H) and the membrane complex V_o_ (containing five subunits, a, d, c, c′ and c″; Kane and Smardon, 2003). V-ATPase-dependent acidification of endosomes is crucial for the entry of several viruses such as HIV, Zika virus and Influenza A (Gruenberg and van der Goot, 2006; Sabino et al., 2019). All this indicates that the endocytic pathway is likely to also have crucial role in the pathogenesis of FV infection.

Here, we aimed to elucidate the role of *Hif-2a* during acute FV infection. Mice with floxed *Hif-2a* (*Hif-2a^fl^*) were resistant to FV infection independent of the expression of Cre recombinase. Hence, we analyzed whether the protection of *Hif-2a*^fl^ mice from FV infection was a direct effect of the loss-of-function of HIF-2α in myeloid cells or a more unspecific effect due to the insertion of the loxP sites.

## RESULTS

### Mice with floxed *Hif-2a* are resistant to FV infection

**Fig. 1.**
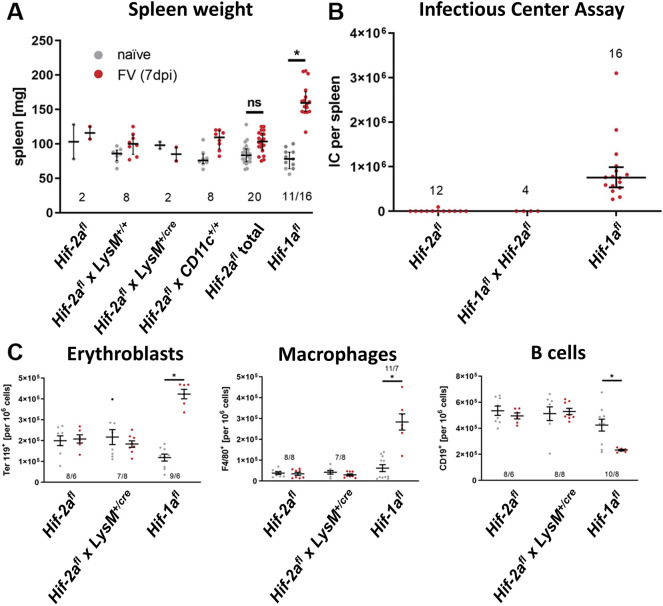
**Mice with a floxed exon 2 of *Hif-2a* are resistant to FV infection.** (A,B) Spleen weight (A) and viral load (B) of different mouse strains were analyzed 7 days after FV infection. Mice designated as *Hif-2a*^*fl*^ in B and C are littermate *Hif-2a*^*fl*^ × *LysM*^+/+^ mice. For reasons of clarity the designation has been shortened. ‘*Hif-2a^fl^* total’ represents the combined results of all different *Hif-2a^fl^* mouse strains. (C) The number of erythroblasts (Ter119^+^), macrophages (F4/80^+^) and B cells (CD19^+^) was determined by flow cytometry. Results are mean±s.e.m. The numbers in the graphs indicate the number of animals tested. *P<0.05; ns, not statistically significant (compared to naïve mice; one-way ANOVA and Tukey's multiple comparison test).

To characterize *Hif-2a* function during acute FV infection, we compared the spleen weights of naïve and FV-infected *Hif-1a^fl^* mice and different *Hif-2a^fl^* mouse strains, as mice that are susceptible to FV-induced disease develop a splenomegaly during acute infection. At 7 days post infection (dpi), the spleen weight of FV-infected *Hif-1a^fl^* mice (here serving as control mice that have the described characteristics of FV infection) by this time had nearly doubled compared to that of healthy, uninfected *Hif-1a^fl^* mice (Fig. 1A). By contrast, none of the *Hif-2a^fl^* mouse strains showed an increase in spleen weight after FV infection. Next, we analyzed the viral loads in the spleens of *Hif-1a^fl^* and *Hif-2a^fl^* mice at 7 dpi with the infectious center assay. Whereas *Hif-1a^fl^* mice showed high viral loads [mean 753,638 infectious centers (IC) per spleen], which were comparable to that of acutely infected C57BL/6 animals (data not shown), significantly lower viral loads were detected in *Hif-2a^fl^* mice (mean 155 IC, Fig. 1B). To determine the role of floxed *Hif-2a*, we crossbred *Hif-2a^fl^* mice with *Hif-1a^fl^* animals. Interestingly, the viral loads in these mice equaled those of the *Hif-2a^fl^* mice (mean 451 IC per spleen), suggesting that the insertion of loxP sites flanking exon 2 of *Hif-2a* also prevents viral loads – and thus FV infection – in *Hif-1a^fl^* mice. To test whether the observed differences between *Hif-1a^fl^* and *Hif-2a^fl^* mice resulted from an elevated or altered immune response, we performed flow cytometry analyses (Fig. 1C). FV infection in *Hif-1a^fl^* mice led to a strong increase in Ter119^+^ erythroblasts due to FV-induced proliferation. Additionally, we observed a pronounced increase in the numbers of F4/80^+^ macrophages in the spleen, accompanied by a significant decrease in CD19^+^ B cells at 7 dpi. However, no differences in the numbers of erythroblasts, macrophages and B cells between naïve and FV-infected *Hif-2a^fl^* and *Hif-2a^fl^*×*LysM^+/cre^* mice were observed. In short, we show that *Hif-2a^fl^* mice are protected from FV infection.

### Spleenoids of *Hif-2a^fl^* mice cannot be infected with FV

**Fig. 2.**
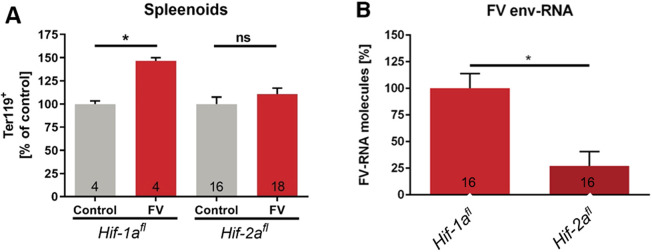
**Resistance of *Hif-2a^fl^* mice persists *in vitro*.** (A,B) Splenic cells from naïve *Hif-1a^fl^* and *Hif-2a^fl^* mice were isolated and cultivated as spleenoids. Spleenoids were infected with FV *in vitro* and analyzed at 4 dpi by flow cytometry (A). Viral load was determined by real time-PCR analysis (B). Expression of *FV envelope* (*env*) RNA was evaluated and normalized to β-actin expression and *Hif-1a^fl^*. Results are mean±s.e.m. The numbers in the graphs indicate the number of animals tested. *P<0.05; ns, not statistically significant (two-tailed unpaired Student's *t*-test).

To further analyze the underlying mechanisms during FV infection in *Hif-2a^fl^* mice, we employed an *in vitro* assay for FV infection using spleenoids. In line with previously published data (Schreiber et al., 2017), a FV infection of spleenoids cultivated from *Hif-1a^fl^* mice resulted in an increase in Ter119^+^ erythroblasts (Fig. 2A). In contrast, a FV infection of spleenoids cultivated from *Hif-2a^fl^* mice did not lead to increased numbers of erythroblasts compared to non-infected control spleenoids. Moreover, real time-PCR analysis showed that FV mRNA expression in *Hif-2a^fl^* spleenoids was more than 75% lower compared to *Hif-1a^fl^* spleenoids (Fig. 2B). Thus, in spleenoids from *Hif-2a^fl^* mice FV infection seems to be tightly controlled.

### *Hif-2a^fl^* mice are not protected from MCMV infection

**Fig. 3.**
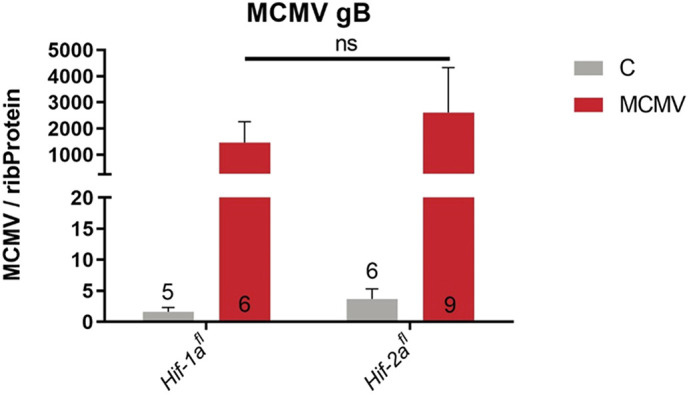
***Hif-2a^fl^* mice show no resistance to MCMV, another dsDNA virus.** BMDMs from naïve *Hif-1a^fl^* and *Hif-2a^fl^* mice were cultivated for 7 days. BMDMs were infected with 10^6^ PFU of MCMV *in vitro* and subjected to mRNA analysis at 2 dpi (data are given as *n*-fold induction of MCMV gB mRNA over mRNA of ribosomal protein 16S (*Rsp16*); C denotes are untreated control cells. Results are mean±s.e.m. The numbers in the graphs indicate the number of animals tested. ns, not statistically significant (two-tailed unpaired *t*-test).

To test whether the *Hif-2a^fl^* mice are resistant to infection by other viruses as well, we chose MCMV (Smith strain), a double-stranded DNA virus, and examined whether bone-marrow-derived macrophages (BMDMs) from *Hif-1a^fl^ and Hif-2a^fl^* are susceptible to MCMV infection *in vitro*. In contrast to our FV infection data *in vivo* and *in vitro,* we detected a similar induction of MCMV glycoprotein B (gB) mRNA in MCMV-infected BMDMs from both genotypes (Fig. 3). The gB protein is an abundant virion envelope protein that is essential for the infectivity of CMV. The increase in MCMV *gB* mRNA was even more pronounced in BMDMs from *Hif-2a^fl^* mice. Therefore, we show that *Hif-2a^fl^* mice are not protected from infection with double-stranded DNA viruses like MCMV.

### MHC I and protein kinase Cε do not contribute to FV resistance of *Hif-2a^fl^* mice

**Fig. 4.**
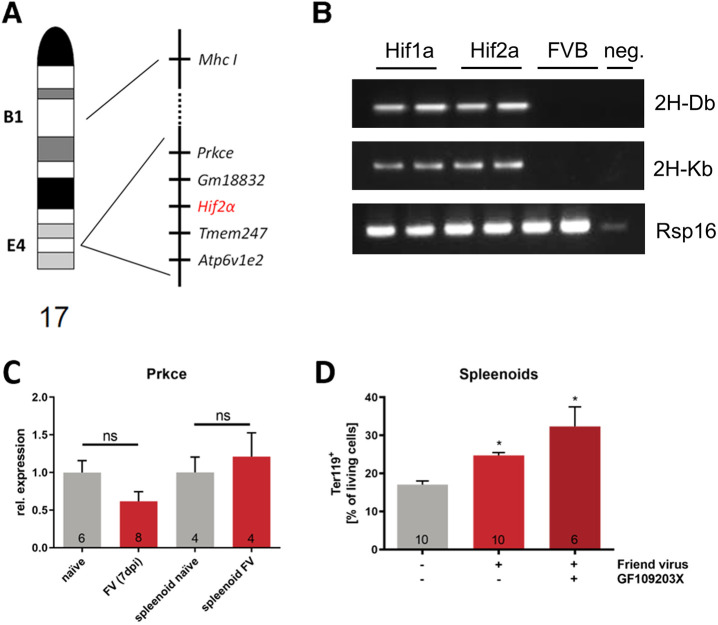
**MHC I and protein kinase C are not involved in FV resistance of *Hif -2a^fl/fl^* mice to FV infection.** (A) Chromosomal structure of the murine chromosome 17. (B) The *Mhc I* haplotype of two individual mice from the *Hif-1a^fl^* and *Hif-2a^fl^* strains was determined by RT-PCR and compared to individuals from the FVB mouse strain as negative control. Image representative of one experimental repeat with two individual mice. (C) To determine whether FV infection alters *Prkce* expression, we performed mRNA expression analyses of naïve and FV-infected *Hif-1a^fl^* mice (7 dpi) and spleenoids (4 dpi). (D) Spleenoids of *Hif-1a^fl^* animals were treated with FV alone or in combination with 0.1 µM of the PRKC inhibitor GF109203X. At 4 dpi, the number of Ter119^+^ erythroblasts was determined by flow cytometry. Results are mean±s.e.m. The numbers in the graphs indicate the number of animals tested. *P<0.05; ns, not statistically significant (two-tailed unpaired Student's *t*-test).

The insertion of loxP sites in the introns flanking exon 2 of *Hif-2a* led to reduced viral loads and thus FV infection *in vivo and in vitro*. As floxed *Hif-2a* is still functional (Gruber et al., 2007), we looked at the chromosomal structure to identify other possible factors involved in FV resistance. Murine *Hif-2a* is located on chromosome 17 (whereas murine *Hif-1a* is located on chromosome 12) in the cytogenetic band E4 (Fig. 4A) in close vicinity of the pseudogene *Gm18832* and the transmembrane protein *Tmem247*, which is expressed solely in testis but without any known function so far (Uhlén et al., 2015). Next to *Gm18832* lies the gene encoding for the ε subunit (*Prkce*) of protein kinase C (PKC), and next to *Tmem247* the *Atp6v1e2* gene encoding for the subunit E2 of the vacuolar ATPase (V-ATPase). Additionally, the gene encoding for the major histocompatibility complex I (*Mhc I*) is positioned in band B1 on chromosome 17 as well. As MHC I plays a major role in FV susceptibility (Lilly et al., 1964; Hasenkrug et al., 1994), we first checked for the haplotype of *Hif-2a^fl^* mice compared to *Hif-1a^fl^* mice. Specific primers for the FV resistance relevant haplotypes H-2D^b^ and H-2K^b^ showed that both strains have the normal C57BL/6 haplotype (Fig. 4B) whereas mice with an FVB background (serving as control samples here) did not express these haplotypes. Next, we looked for an involvement of *Prkce*, as other retrovirus entry receptors are regulated by PKCε (Jobbagy et al., 1999; Gräf et al., 2001). Expression analyses of *Prkce* in FV-infected *Hif-1a^fl^* mice and spleenoids showed no differences to naïve controls (Fig. 4C). To determine whether *Prkce* is involved in FV infection, we used the PKC inhibitor GF109203X. Inhibition of PKC did not alter FV-induced erythroblast proliferation in spleenoids (Fig. 4D). Taken together, we show that neither *Mhc I* nor *Prkce* seem to be involved in resistance against FV infection.

### *Hif-2a*^fl^ mice showed a reduced expression of the V-ATPase subunit E2

**Fig. 5.**
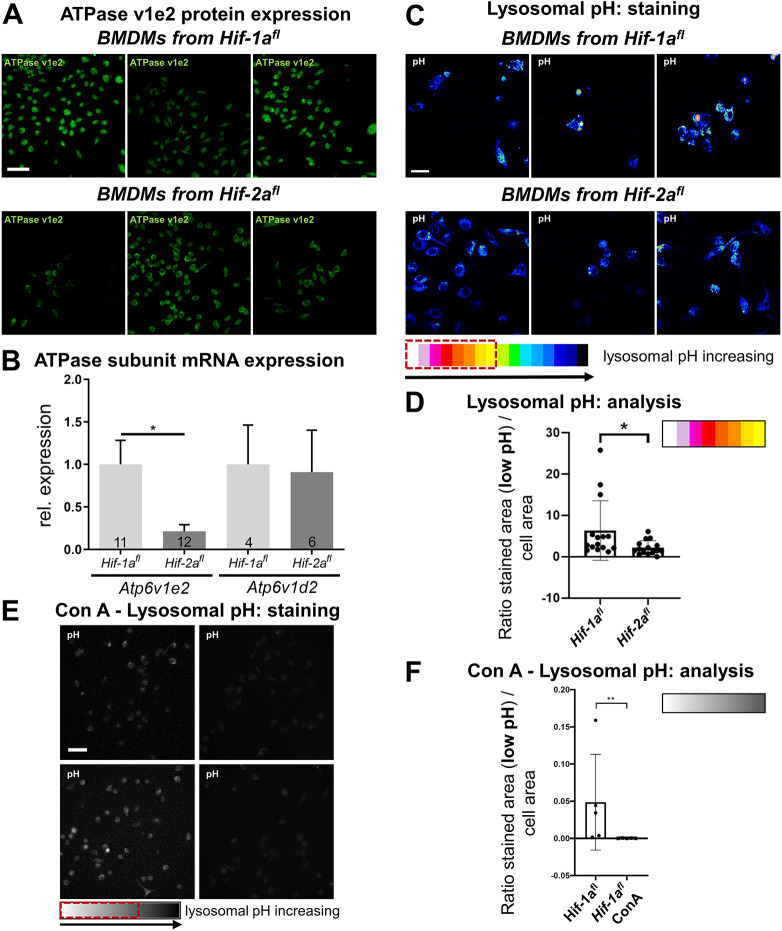
**BMDMs from *Hif -2a^fl^* mice show a reduced expression of the V-ATPase subunit E2 and show a reduced acidification of lysosomes.** (A,B) The V-ATPase subunit E2 was immunocytochemically stained in BMDMs from *Hif-1a^fl^* and *Hif-2a^fl^* mice. (A) Three representative images from the five independent mice with floxed *Hif-1a* or *Hif-2a* that were analyzed are shown. (B) Relative mRNA expression of *Atp6v1e2* and *Atp6v1d2* in BMDMs from *Hif-1a^fl^* and *Hif-2a^fl^* mice. Results are mean±s.e.m. *P<0.05 (two-tailed unpaired Student's *t*-test). (C,D) The lysosomal pH was examined with LysoSensor™, with increasing yellow to red coloring indicating a lower lysosomal pH. (C) Three representative images from the three independent mice with floxed *Hif-1a* and *Hif-2a* that were analyzed are shown. (D) The numbers of marked (red dotted box in C) yellow to white and white pixels color representing acidification within the cell was counted for 15 representative images from each of three independent mice with floxed *Hif-1a* and *Hif-2a.* A ratio of these pixels to the total stained area was calculated and is presented as mean±s.d. Floxed *Hif-2a* mice showed a significantly decreased acidification of lysosomes. **P<0.01 (Mann–Whitney test). A and C are representative images of cells from only one mouse per strain. For representative images of all analyzed individuals please refer to Fig. S1. (E,F) The lysosomal pH was in BMDMs from *Hif-1^fl^* mice was examined as in C, with control BMDMs shown in the left two pictures and ConA-treated BMDMs shown in the right two pictures. *n*=5 pictures from three independent mice for each group. (F) The marked (see red dotted box in E) white to grey numbers of pixels was counted for five representative images from BMDMs of three independent floxed *Hif-1a* mice with and without ConA treatment. A ratio of these pixels to the total stained area was calculated and is shown as mean±s.d. ConA-treated cells showed a significantly decreased acidification of lysosomes. **P<0.01 (Mann–Whitney test). Scale bars: 40 μm (A), 20 μm (C,E).

The *Atp6v1e2* gene is also located on chromosome 17 and could therefore be affected by the insertion of the loxP sites flanking exon 2 of the *Hif-2a* gene. As the vacuolar ATPase subunit is involved in viral entry (Guinea and Carrasco, 1995), we investigated whether this enzyme plays a role in protecting our *Hif-2a^fl^* mice from FV infection. To determine the expression levels of the V-ATPase subunit E2 in *Hif-2a^fl^* mice, we used immunocytochemical stainings to analyze the cellular distribution in BMDMs. We observed a substantially reduced expression of the V-ATPase subunit E2 in BMDMs of *Hif-2a^fl^* mice compared to the found in *Hif-1a^fl^* BMDMs (Fig. 5A). Additionally, we found a significantly lower abundance of the mRNA of the subunit *Atp6v1e2* in *Hif-2a^fl^* animals compared to that in *Hif-1a^fl^* mice (Fig. 5B) and also compared to that in *Fih^fl^* mice on C57BL/6 background, which we bred in our animal facility (data not shown). Cre expression did not influence the mRNA abundance of *Atp6v1e2* as BMDMs from *Hif-2a^fl^* and *Hif-2a^fl^*×*LysM^+/cre^* mice did not show any differences in the expression of *Atp6v1e2* (Fig. S2). Sequencing of the complete coding sequence of the *Atp6v1e2* DNA from both genotypes showed no differences between *Hif-1a^fl^* and *Hif-1a^fl^* ×*Hif-2a^fl^* mice (Fig. S3).

### The reduced expression of the V-ATPase subunit E2 resulted in an elevated lysosomal pH

The insertion of loxP sites flanking exon 2 of the *Hif-2a* gene is ∼147 kilobase pairs (kbp) away from the *Atp6v1e2* gene (see Fig. S4) but nevertheless caused a suppression with functional consequences in macrophages. Interestingly, the mRNA expression of *Atp6v1e2* was unaltered in splenic T and B cells from *Hif-2a^fl^* compared to *Hif-1a^fl^* mice (Fig. S5). In BMDMs, an analysis of the intralysosomal pH revealed that insertion of the loxP sites resulted in reduced V-ATPase subunit E2 levels (Fig. 5A,B) and affected the lysosomal pH. We detected a significantly higher pH in the lysosomes of unstimulated BMDMs isolated from *Hif-2a^fl^* mice compared to the lysosomal pH in BMDMs from *Hif-1a^fl^* mice (Fig. 5C,D). Treatment of macrophages isolated from *Hif-1a^fl^* mice with concanamycin A (ConA), as a highly specific inhibitor of the vacuolar ATPase, resulted in a significantly elevated lysosomal pH and therefore prevented a lysosomal acidification (Fig. 5E,F).

Taken together, we show a significant reduction of both the *Atp6v1e2* mRNA and protein expression of vacuolar ATPase subunit E2 in *Hif-2a^fl^* mice and a reduction of the acidifying function of the vacuolar ATPase in these mice.

### A reduced expression of the V-ATPase subunit E2 leads to a reduced virulence of FV

**Fig. 6.**
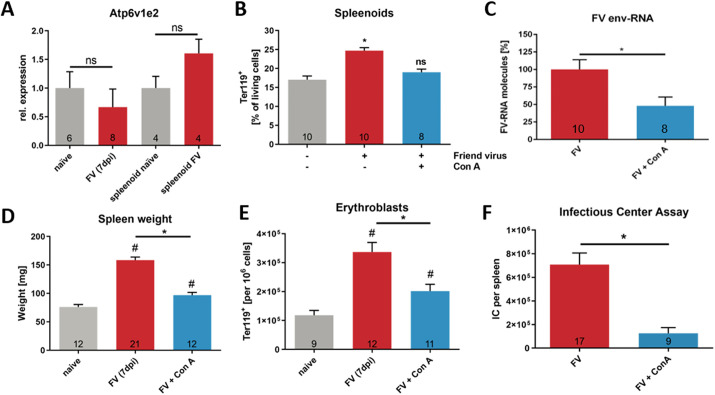
**Disturbed V-ATPase function leads to reduced virulence of FV.** (A) To determine whether V-ATPase expression is altered during FV infection, expression analyses of the subunit *Atp6v1e2* of naïve and FV infected *Hif-1a^fl^* mice (7 dpi) and spleenoids (4 dpi) were performed. (B,C) Spleenoids were treated with FV either alone or in combination with 1 µM ConA. (B) At 4 dpi, the number of erythroblasts (Ter119^+^) was determined by flow cytometry. (C) Viral load was determined by real time-PCR analyses for *FV env* expression. Expression was normalized to β-actin expression. (D) Spleen weight of *Hif-1a^fl^* mice with and without ConA treatment (12 ng/g body weight) was analyzed 7 days after FV infection. (E) The numbers of erythroblasts (Ter119^+^) in spleens of *Hif-1a^fl^* mice with or without ConA treatment was determined by flow cytometry. (F) Viral load of *Hif-1a^fl^* mice with or without ConA treatment was analyzed at 7 dpi. Results are mean±s.e.m. The number of animals tested/experiments is indicated in the graphs. *P<0.05; ^#^*P*<0.05 compared to naïve mice; ns, not statistically significant (one-way ANOVA and Tukey's multiple comparison test).

Expression analyses showed no differences in *Atp6v1e2* mRNA between naïve and FV-infected *Hif-1a^fl^* mice and spleenoids (Fig. 6A). However, inhibition of the V-ATPase using the highly specific inhibitor ConA at a nanomolar concentration during FV infection in spleenoids cultured from *Hif-1a^fl^* mice resulted in reduced Ter119^+^ erythroblast proliferation and viral replication (Fig. 6B,C). Next, we studied the involvement of V-ATPase in FV infection *in vivo*. For this, FV susceptible *Hif-1a^fl^* mice were treated with 12 ng ConA per g of body weight at 1 day prior to FV infection and consecutively every second day. The spleen weight of naïve and FV-infected mice as well as those of mice that were treated with ConA before and during the FV infection were analyzed at 7 dpi. The increase in spleen weight caused by FV infection was significantly reduced by the treatment with ConA, and the spleen weights after ConA treatment were similar to those of naïve controls (Fig. 6D). Additionally, the increase in erythroblast proliferation was almost absent after treatment with ConA (Fig. 6E), and finally, viral loads in the spleen were significantly reduced in mice treated with Con A (Fig. 6F). Taken together, we show that the inhibition of the V-ATPase resulted in decreased viral loads in infected mice potentially due to arrested vesicular trafficking. Thus, we conclude (1) that a functional V-ATPase is indispensable for FV infection and (2) that the insertion of loxP sites flanking exon 2 of the *Hif-2a* gene in *Hif-2a^fl^* and *Hif-1a^fl^×Hif-2a^fl^* mice results in reduced expression and function of V-ATPase preventing the infection with FV.

## DISCUSSION

FV infections are well characterized with respect to the susceptibility of different mouse strains. Whereas susceptibility to FV (seen in e.g. BALB/c mice) results in a lethal erythroleukemia and splenomegaly, resistant mice (e.g. on C57BL/6 background) get infected and develop a moderate splenomegaly during acute infection. Resistant mice can partially recover from the infection due to their strong immune answer and develop a persistent, but controlled, infection (Kumar and Bennett, 1981). Here, we show that functional V-ATPase is required for efficient FV infection and that genetic manipulation of C57BL/6 mice by the insertion of loxP sites flanking exon 2 of the *Hif-2a* gene is able to reduce the expression of the V-ATPase subunit E2 (Fig. 5A,B). This reduces the acidification of endosomes and therefore protects mice and spleenoids from FV infection (Fig. 5C,D).

The spleen weight and the viral load were unchanged in both *Hif-2a^fl^* and *Hif-2a^fl^*×*LysM-Cre^+/cre^* animals (Fig. 1A,B) after acute infection with the FV. In addition, the mice showed neither erythrocythemia (Fig. 1C) nor changes in splenic macrophage or B cell numbers (Fig. 1D,E). In short, they were protected from FV infection, which was completely unexpected. We found that neither the expression of Cre recombinase nor its control by different promoters (Lyz2 Cre or Itgax Cre, see Fig. 1A) affected the infection. We conclude that myeloid knockout of HIF-2α is not responsible for this effect as *Hif-2a* knockdown BMDMs showed an identical expression of *Atp6v1e2* mRNA compared to BMDMs from *Hif-2a^fl^* mice (Fig. S2) but that the insertion of the loxP sites around exon 2 of the *Hif-2a* gene must be responsible for this finding. This is corroborated by the fact that the genetic background of *Hif-1a^fl^* and *Hif-2a^fl^* mice is identical (see Materials and Methods). Thus, specific insertion of loxP sites around exon 2 of the *Hif-2a* gene caused the phenotype.

A resistance to infection with ecotropic MuLV has been described previously for mice expressing very high amounts of the *Fv4* gene (Limjoco et al., 1993). Those authors detected no viral loads in spleens and blood of FV-infected animals at 21 to 38 dpi. *Fv4* encodes an endogenous MuLV envelope protein that causes resistance to infection by exogenous ecotropic murine retroviruses like FV (Kai et al., 1976). In this context, the reduction of virus entry and infection is dependent on the level of *Fv4* locus expression and is not absolute (Taylor et al., 2001). Mice carrying the dominant allele of *Fv4* (corresponding to the defective provirus) are resistant to infection with ecotropic MuLVs because they express a defective Env protein that blocks virus interference with its receptor (Ikeda and Sugimura, 1989). We compared data on these animals to that of *Hif-1a^fl^* animals, as we knew that these mice could be infected with FV *in vivo* (Schreiber et al., 2017) and that the *Hif-1a* gene is located on chromosome 12 in proximity to the *Fv4* gene. *Hif-1a*^*fl*^ mice showed normal values of viral load, erythrocythemia and immune cell numbers after infection comparable to other C57BL/6 animals that can control the initial infection and recover from it (Dittmer et al., 2019). Erythrocythemia only occurred in *Hif-1a^fl^*, but not in *Hif-2a^fl^* animals (Fig. 1D). As *Hif-1a^fl^* mice showed normal characteristics of FV infection and the insertion of loxP sites occurred in chromosome 17 in the *Hif-2a^fl^* mice we did not further analyze an influence of *Fv4* on the FV resistance of our *Hif-2a^fl^* and *Hif-2a^fl^×LysM-Cre^+/cre^* animals.

Consistent with the *in vivo* experiments, FV could not infect spleenoids from *Hif-2a^fl^* animals *in vitro*; thus, the resistance persists in cultivated cellular organoids developed from isolated murine cells. Neither a proliferation of erythroblasts (Fig. 2A) nor the viral load (Fig. 2B) changed significantly after FV infection of spleenoids cultivated from *Hif-2a^fl^* mice, which differs strongly from what is seen with *Hif-1a^fl^* mice spleenoids. These data recapitulate that from previous experiments where we observed very similar results in FV-infected spleenoids from *Hif-1a^fl^* mice (Schreiber et al., 2017). The erythrocythemia in spleenoids from *Hif-1a^fl^* mice is most likely due to increased erythropoiesis as the mouse spleen is able to perform erythropoiesis analogously to the bone marrow (Metcalf et al., 1959; Brodsky et al., 1966).

It has been published that the enzymatic activity of protein kinase C is essential for erythroid precursor cell proliferation whereas a decrease in enzyme activity within the nucleus is associated with differentiation of these cells (Beckman, 1992). In addition, protein kinase Cε regulates the expression and transport activity of retroviral receptors in murine and human cells (Jobbagy et al., 1999; Gräf et al., 2001). We did not find significant differences in *Prkce* mRNA expression between naïve and FV infected *Hif-1a^fl^* mice (Fig. 4C, left part) or between *in vitro* FV-infected and uninfected spleenoids from *Hif-1a^fl^* animals. Furthermore, treatment of these spleenoids with the PKC inhibitor GF109203X during FV infection *in vitro* did not prevent the proliferation of Ter119^+^ erythroid progenitor cells as we observed it to do in the *Hif-2a^fl^* mice. This suggests that potential modifications of either the *Mhc I* or the *Prkce* gene by the loxP sites flanking exon 2 of the *Hif-2a* gene are unlikely to protect these mice from FV infection.

The induced resistance in *Hif-2a^fl^* mice is restricted to FV infection as the double-stranded DNA virus MCMV is detectable in comparable amounts in BMDMs from *Hif-1a^fl^* and *Hif-2a^fl^* mice that were infected *in vitro* (Fig. 3). This is in line with a previous report (Scrivano et al., 2010) in which the authors observed MCMV infection to be independent of bafilomycin A treatment. Bafilomycin A, like ConA, is an inhibitor of the vacuolar ATPase. Furthermore, the entry of human CMV (HCMV) depends on the cell type. Infection of fibroblasts (Compton et al., 1992) and dendritic cells (Haspot et al., 2012) has been shown to be pH independent, whereas HCMV entry into epithelial and endothelial cells is pH dependent (Bodaghi et al., 1999; Ryckman et al., 2006). In contrast to HCMV, many viruses, like Dengue virus, Zika virus, rhinovirus, influenza virus A and VSV, enter host cells via pH-dependent mechanisms, which require lysosomal acidification by proton translocation into the lysosomal lumen by the vacuolar ATPase (Marjuki et al., 2010; Ho et al., 2017; Sabino et al., 2019; Suzuki et al., 2001; Li et al., 2020; Müller et al., 2011). In addition, pH-dependent endocytic pathways for infection have been observed for ecotropic and amphotropic MuLVs (Katen et al., 2001). Other retroviruses, like HIV-1 or -2 or human T-cell leukemia virus type 1 (HTLV-1) or 2, enter the cells pH independently, indicating that vacuolar ATPase is not required for HIV or HTLV entry (McClure et al., 1988; Stein et al., 1987). Interestingly, a recent report has suggested that the V_o_ subunit C of the vacuolar ATPase, which forms the proton transporting channel, plays a role in regulating tetherin expression and therefore regulates the assembly and release of HIV (Waheed et al., 2020). Thus, it seems that apart from its role in viral entry by lysosomal acidification, the vacuolar ATPase is also important for the release of newly synthesized virions. The latter is regulated via the expression of the restriction factor tetherin (Liberatore and Bieniasz, 2011), which has also been shown to play an important role in FV immunity (Li et al., 2014, 2016).

Interestingly, we found a significantly reduced expression of the V-ATPase subunit E2 in BMDMs from *Hif-2a^fl^* mice compared to that in *Hif-1a^fl^* animals (Fig. 5A for protein; Fig. 5B for mRNA). This subunit of the V-ATPase is an anchor for the C subunit that links the V_1_ and V_o_ complex in a functional enzyme. Disassembly of V_1_ and V_o_ leads to loss-of-function and acidification of the lysosome ceases (Kane and Smardon, 2003; Hayek et al., 2019). This is the reason why Grueber et al. (Grüber et al., 2002) emphasize that the subunit E is essential for ATPase function. They conclude that shielding of this subunit in the inner part of the enzyme preserves enzymatic function. Sequencing of the coding region of the *Atp6v1e2* gene did not reveal any differences from the known sequence for this gene (Fig. S3), which underlines our result that it is a decrease in the expression of the *Atp6v1e2* gene and not a loss-of-function mutation within the gene. As the distance between the loxP sites flanking exon 2 of *Hif-2a* and the *Atp6v1e2* gene is 147 kbp (see Fig. S4), we propose that indirect effects are responsible for the specific decrease in the *Atp6v1e2* gene expression after insertion of the loxP sites. The region around *Hif-2a* exon 2 might function as an enhancer of *Atp6v1e2* transcription. A known enhancer (ID: ENSMUSR00000128964) lies on chromosome 17 in the region of 87.100.202–87.111.468 bp, overlapping with exon 2 of *Hif-2a* (Martin et al., 2023). This enhancer region is active in the adult murine spleen and is associated with different proteins (e.g. histone H3 and the CCCTC-binding factor CTCF) that regulate the 3D structure of chromatin and therefore, gene regulation. However, to our knowledge there is no prior evidence that this enhancer is associated with the expression of *Atp6v1e2*. Regarding the functional consequences of the downregulation of *Atp6v1e2* by the insertion of loxP sites (i.e. the higher lysosomal pH values seen in Fig. 5C,D), we conclude that vacuolar acidification is crucial for FV infection of murine cells. Interestingly, T and B cells of the spleens of *Hif-2a^fl^* mice did not show a reduced expression of the *Atp6v1e2* mRNA (Fig. S5). There are some possible explanations for this finding. First, it is not known if the enhancer, located in exon 2, is active in lymphocytes. Second, the V_1_ domain of the ATPase contains tissue-specific subunit isoforms including B, C, E and G. Therefore, the E2 subunit *Atp6v1e2* encodes for might not be of importance in lymphocytes. And third, the expression of *Hif-2a* is substantially lower in B and T cells compared to in BMDMs, which might influence the expression of the *Atp6v1e2* [ΔCt=Ct(*Hif-2a*)−Ct(*Rps16*): BMDMs, 10.74±0.81; B cells, 12.13±1.16; T cells, 13.44±1.34; mean±s.d. of 10 mice per group; data not shown]. All in all, *Hif-2a^fl^* mice do not show any signs of FV infection, although there is no change in the expression of *Atp6v1e2* in cells other than macrophages. This might be due to the fact that the injected viral titers were too low to infect lymphocytes without prior replication in macrophages. Honke et al. have already described an enforced viral replication by macrophages in murine VSV and lymphocytic choriomeningitis virus (LCMV) infection. Hence, this mechanism that might be worth investigating during FV infection as well (Honke et al., 2011). Of note, T cells are not considered the main target of FV, and B cells have been shown to be infected at later time points compared to myeloid cells (Windmann et al., 2019), which points to a previously undescribed role for the vacuolar ATPase in macrophages during FV spread. Supporting this notion, inhibition of the vacuolar ATPase by ConA was able to prevent the increase in erythroid precursor cells in FV-infected spleenoids of *Hif-1a^fl^* mice. Administration of ConA prior to *in vivo* FV infection almost completely impeded signs of FV infection with respect to splenomegaly and viral load (Fig. 6D–F). Franchini et al. (1993) found a direct interaction of the HTLV-1 p12 protein with the vacuolar H^+^ ATPase. They pointed out regions of high-level amino acid conservation between HTLV-1 p12 and Friend-SFFV1 (spleen focus-forming virus, the part of the FV complex, which conveys the polyclonal proliferation of erythroblasts and therefore the erythroleukemia) and -SFFV2 (Franchini et al., 1993). Therefore, a direct interaction between FV and the H^+^-ATPase seems possible and interesting to analyze in further studies. Whether the interference of the inserted loxP sites with the function of the V-ATPase is responsible for other findings in other studies that have used this particular mouse strain will be hard to interpret, as the V-ATPase plays an important role in a variety of different biological processes, like toxin delivery, membrane targeting, apoptosis, regulation of cytoplasmic pH, proteolytic process, acidification of intracellular systems, autophagy and many more (Eaton et al., 2021; Kenney and Benarroch, 2015). In addition, unspecific effects due to the insertion of the loxP sites alone will be immediately unraveled as they would also manifest in Cre-negative siblings. This corroborates the need for suitable control animals in animal studies. To the best of our knowledge, this strategy has excluded so far that the reduced expression of V-ATPase might be responsible for findings that have since then been alluded to HIF-2α deficiency.

Our data are based on the use of ConA, which although a highly specific inhibitor of the vacuolar ATPase is not, for example, a direct knockdown of the *Atp6v1e2* gene; this has to be taken into account to prevent an overinterpretation of the findings presented herein. This is due to the fact that our initial focus was on the role of myeloid *Hif-2a* in FV infection.

Collectively, these findings demonstrate that the insertion of loxP sites flanking exon 2 of the *Hif-2a* gene on chromosome 17 results in a significant decrease in the expression of the *Atp6v1e2* gene and in a reduced protein abundance of the E2 subunit of vacuolar ATPase. This in turn might block the assembly of the V-ATPase subunits V_1_ and V_o_ and therefore hamper the acidification of lysosomes. Consequently, this mechanism might protect *Hif-2a^fl^* mice from infection with FV.

## MATERIALS AND METHODS

### Animals

*Hif-1a* (*Hif-1a^fl^,* stock number #007561) and *Hif-2a* (*Hif-2a^fl^*, stock number #008407) mice were purchased from The Jackson Laboratory, Bar Harbor, ME, USA. Both mouse strains have initially been established via 129X1/SvJ-derived embryonic stem cells and have then been crossbred to C57BL/6 mice. In detail, these mice exhibit loxP sites flanking exon 2 of the *Hif-1a* (*Hif-1a^fl^*) or the *Hif-2a* (*Hif-2a^fl^*) gene on both alleles. The mice were crossbred with mice carrying a Cre recombinase either under the control of a lysozyme 2 gene (*Lyz2*) promotor (*LysM*) or an integrin subunit αX gene (*Itgax*) promotor (corresponding to *CD11c*) (see Table S1).

Exon 2 encodes for the DNA-binding domain of the translated HIF-1α or -2α protein. Littermates negative for Cre recombinase served as control (wild-type) mice. We also used *Hif-2a^fl^* from a separate breeding line (*Hif-2a^fl^* in Fig. 1A) to exclude any breeding artefacts in our mouse colony*.* For some analyses, we also used *Hif-1a^fl^*×*Hif-2a^fl^* mice. These behaved exactly like *Hif-2a^fl^, Hif-2a^fl^*×*LysM^+/+^* and *Hif-2a^fl^*×*CD11c^+/+^* animals with respect to FV infection (Fig. 1B)*.* Mice with the FVB background were used to compare the MHC I haplotype to that of *Hif-1a^fl^* and *Hif-2a^fl^* strains on C57BL/6 background (Fig. 4B). All animal experiments were performed in full accordance with the German law for animal welfare and with institutional regulations for animal breeding and handling, and were approved by the State Agency for Nature, Environment and Consumer Protection North Rhine-Westphalia (file reference: 84-02.04.2013.A317). All animals behaved and bred normally.

### Bone marrow-derived macrophages

BMDMs were isolated from *Hif-1a^fl^* and *Hif-2a^fl^* mice and cultivated as previously described (Schreiber et al., 2017; Kerber et al., 2020). Animals that were not subjected to an experiment before were killed by cervical dislocation for a scientific purpose according to Section 4.3 of the German law for animal welfare. Briefly, the bone marrow was flushed from the bone cavity with a 23 G needle and syringe (BD Biosciences; Heidelberg, Germany) containing macrophage medium [minimal essential medium, MEM, plus 100 U/ml penicillin/streptomycin, 5 mM L-glutamine, 1% (v/v) sodium pyruvate (all from Invitrogen, Waltham, MA, USA), 1 mM HEPES (Sigma-Aldrich, Munich, Germany) and 0.6 mM tissue culture grade β-mercaptoethanol (Merck KGaA, Darmstadt, Germany) and 10% (v/v) fetal calf serum (FCS; Biochrom GmbH, Berlin, Germany)]. Bone marrow flush cells were seeded in cell culture flasks (T-25; BD Biosciences; Heidelberg, Germany) in 7 ml of macrophage medium. After 24 h of incubation at 37°C and 5% CO_2_, the non-adherent population was transferred into a six-well culture plate (Sarstedt, Nuembrecht, Germany) at a density of 10^6^ cells per well with fresh macrophage medium containing 30% L929 cell-conditioned medium (produced in our laboratory under sterile conditions, M-CSF concentration: 100 pg/ml). The medium was renewed every second day and after an additional 5 days in culture, the adherent BMDMs were harvested for experiments.

### Viral infections

FV stocks were produced as previously described (Schreiber et al., 2017). Briefly, BALB/c mice were infected 14 days earlier with 3000 spleen focus-forming units (SFFUs) of non-cloned virus and the experimental virus stock was prepared as a 10% spleen cell homogenate (in PBS) from these mice. Experimental mice (male and female mice, 8–16 weeks at the beginning of the infection) were injected intravenously (i.v.) with 0.1 ml phosphate-buffered saline (PBS) containing 20,000 SFFU of FV. The virus stock was free of lactate dehydrogenase-elevating virus. Animals were killed at 7 days post infection (dpi).

Bone marrow-derived dendritic cells for *in vitro* murine cytomegalovirus (MCMV) infection were isolated from bone marrow of the hind limbs of *Hif-1a^fl^* and *Hif-2a^fl^* mice. The bones were prepared and opened in a sterile manner, and the bone marrow was flushed out with a G 27×3/4″ cannula. The cell suspension was stained with Trypan Blue and 10^6^ living cells were seeded into each well of a six-well plate in RPMI 1640 medium (10% FCS, 2 mM glutamine, 1 mM sodium pyruvate, 100 U/ml penicillin, 100 µg/ml streptomycin, 0.05 mM 2-mercaptoethanol) containing 1 ng/ml of murine IL-4 and 5 ng/ml of murine GM-CSF. Half of the medium was renewed every second day and after 7 days cells were infected with 10^6^ plaque-forming units (PFU) of MCMV (Smith strain; kindly provided by Prof. Mirko Trilling, University Hospital Essen). At 48 h later the cells were lysed with 4 M guanidinium thiocyanate (GTC) and the cellular RNA was isolated as described below.

### ConA treatment of mice

To test the involvement of the V-ATPase in FV infection, we injected 12 ng per g of body weight of the V-ATPase inhibitor Concanamycin A (ConA; Santa Cruz Biotechnology, Heidelberg, Germany, and Sigma-Aldrich, Munich, Germany) intraperitoneally (i.p.) (in 50 µl sterile PBS) 1 day prior to FV infection and consecutively every second day. The animals were killed by cervical dislocation at 7 dpi.

### Spleenoid culture

Spleenoids were cultivated as previously described (Schreiber et al., 2017). Briefly, we generated single-cell suspensions from spleens and diluted them with PBS to a concentration of 10^8^ cells per ml. For each spleenoid, we centrifuged 10 µl of the cell suspension and the cells were resuspended in ice-cold Matrigel (BD Biosciences, Heidelberg, Germany). The Matrigel/cell droplets were allowed to gel at 37°C and were subsequently grown in spleenoid medium (Aim-V medium; Thermo Fisher Scientific, Darmstadt, Germany) supplemented with 10% FCS, 4 mM L-glutamine, 0.6 mM tissue culture grade β-mercaptoethanol, and 100 U/ml penicillin and 100 µg/ml streptomycin). After 3 days of stationary growth, the tissue droplets were infected with FV, transferred into a spinning bioreactor and cultivated for an additional 4 days. For ConA and GF109203X (PKC inhibitor; Bio-Techne, Wiesbaden, Germany) treatment, the spleenoids were treated with the respective substance 24 h prior to FV infection.

### Infectious center assay

The extent of the viral infection was determined with an infectious center assay according to this previous description (Dittmer et al., 1998). Briefly, titrations (10^7^–10^2^ cells/ml) of single-cell suspensions from infected splenocytes were seeded onto susceptible *Mus terricolor* cells (ATCC #CRL-2017), co-cultivated for 5 days, and stained with F-MuLV envelope-specific monoclonal antibody 720 (Robertson et al., 1991) and horseradish-peroxidase coupled goat anti-mouse-IgG (Jackson ImmunoResearch, West Grove, PA, USA) to detect foci. Note, the assay is performed in undiluted supernatants of hybridoma cells that contain an unknown concentration of the 720 antibody. The volume of the supernatant is adjusted for each new batch of supernatant stock to ensure a comparability of results.

### Cell-surface staining by flow cytometry

Spleen cells were stained as previously described (Schreiber et al., 2017) with fluorochrome-conjugated antibodies and analyzed with a FACSCalibur or FACSCelesta flow cytometer and the FACSDiva software (BD Biosciences, Oxford, UK). Cells were stained with the following antibodies [each diluted 1:200 in FACS buffer (PBS containing 0.1% FCS and 0.02% NaN_3_)]: anti-Ter119 (TER-119; eBioscience, Inc., San Diego, CA, USA), anti-F4/80 (CI:A3-1, Biolegend, San Diego, CA, USA), anti-CD19 (Cl:6D5; Biolegend) and anti-CD3 (Cl:17A2, Biolegend). Dead cells were excluded with the use of a fixable viability dye (FVD)-eFluor 780 (eBioscience) staining.

### Magnetic-activated cell sorting

T and B cells were isolated from spleens of naïve mice by negative selection using a pan T cell isolation kit II and pan B cell isolation kit, respectively (Miltenyi Biotec), according to the manufacturer's instructions. Red blood cells from spleens were lysed prior to MACS isolation. The purity of isolated cell populations was assessed by flow cytometry. The percentage of positively labeled cells within the isolated population reached >90% for both T and B cell fractions (see Fig. S5D,E).

### RNA isolation and PCR analyses

RNA isolation and PCR analyses were performed as previously described (Schreiber et al., 2017). The amounts of cDNA were normalized to the expression of ribosomal protein (*Rsp16*) or β-actin (*Actb*) as indicated in the respective figure legend and the relative expression was calculated with the 2^−ΔΔCt^ method. The following primers (all 5′–3′) were used: *Hif-2a* exon 2 sense, AGGAGACGGAGGTCTTCTATGA; *Hif-2a* exon 2 antisense, ACAGGAGCTTATGTGTCCGA; *H-2D^b^* sense, AGTGGTGCTGCAGAGCATTACAA; *H-2d^b^* antisense, GGTGACTTCACCTTTAGATCTGGG; *H-2K^b^* sense, GAGCCCCGGTACATGGAA; *H-2K^b^* antisense, CAGGTAGGCCCTGAGTCT; *Prkce* sense, GCTCCTATCGGCTACGACG; *Prkce* antisense, CCAGGTCAATCCAGTCCTCG; *Atp6v1e2* sense, ACCATGAGAAACCAGGCTCG; *Atp6v1e2* antisense, GATCGCTGCTGTAGACCTCC; *Atp6v1d2* sense, CTGGTTCGAGGATGCAAAGC, *Atp6v1d2* antisense, TTGCCATAGTCCGTGGTCTG, *Rsp16* sense, TACTGGAGCCTGTTTTGCTTCT; *Rsp16* antisense, CCCTTCACACGGACCCGAAT; *Actb* sense, ATATCGCTGCGCTGGTCG, *Actb* antisense, TTCCCACCATCACACCCTGG; *MCMV gB* sense, TGGCGACTGCTAGCCCTCCT; *MCMV gB* antisense, GTCGCGACACCAAGCGTCCA; and *FV*.*env* sense, ACTTATTCCAACCATACCTCT, *FV.env* antisense, TTTAGCTGGTGGTATTGTTGA (Smith et al., 2011).

### Immunocytochemistry

We cultured BMDMs in 24-well plates at a density of 250,000 cells in 250 μl medium for the immunocytochemistry experiments. The medium was discarded and the cells were fixed and permeabilized with 250 μl ice-cold acetone/methanol (1:1) at −20°C for 10 min. The solvent was then discarded, and the cells dried at room temperature (10 min). 3% BSA in PBS was then used for blocking before the antibody incubation with anti-ATP6V1E2 (#PA5-29206, Thermo Fisher Scientific, Darmstadt, Germany; 1:200 in 3% BSA in PBS) and an AlexaFluor 488-labeled secondary antibody (goat anti-rabbit IgG Alexa Fluor 488 antibody, #A-32731, Thermo Fisher Scientific; 1:200 in PBS) were used. Images were taken at a confocal microscopy system (LSM 500, Zeiss, Jena, Germany). All images have been processed with the ZEN system 2012 software (blue edition; Zeiss).

### Analysis of lysosomal pH

The lysosomal pH was analyzed with the help of the LysoSensor™ Blue DND-167 dye (#L7533, Thermo Fisher Scientific). This LysoSensor dye is quenched by alkaline environments. The pK_a_ of LysoSensor Blue DND-167 is ∼5.1. BMDMs from *Hif-1a^fl^* and *Hif-2a^fl^* mice have been incubated with 1 µM LysoSensor for 30 min and were then directly analyzed with the Light Microscopy ELYRA or AxioObserver.Z1 (Zeiss). Images were processed with ZEN system 2012 (black edition; Zeiss) and ImageJ Fiji software (Schindelin et al., 2012). Positive staining is indicated by the respective red boxes in Fig. 5C,E. The experiment shown is representative for two individually repeated measurements (with eight mice in total).

### Sanger sequencing

We isolated genomic DNA from ear punching tissue of *Hif-1a^fl^* and *Hif-1a^fl^*×*Hif-2a^fl^* mice (which behaved exactly like *Hif-2a^fl^* with respect to FV infection, see Fig. 1B). The tissue was incubated in 50 mM NaOH at 100°C for 1 h, then Tris-HCl (1 M) was added to the samples and those were mixed well. Afterwards, a centrifugation step (1957 ***g*** for 2 mins) removed any tissue remnants. The primers used for PCR were as follows: sense, 5′-ACATTGAAGACACCAGAAGG-3′, antisense, 5′-GAAGGCTCTGCAGTTTAGCC-3′. The primer pair spans the whole protein coding region of the *Atp6v1e2* gene. Afterwards PCR products of the samples were processed with the DNA Clean & Concentrator™-Kit (Zymo Research, Freiburg, Germany). Samples were further processed as directed by Microsynth Seqlab (Göttingen, Germany) who performed the Sanger Sequencing (sequencing primer sequence 5′-ACATTGAAGACACCAGAAGG-3′). Results were viewed and depicted with SnapGene software (Version 5.1.7; GSL Biotech LLC, Chicago, IL, USA).

### Statistics

Data were analyzed with one-way ANOVA and Tukey's multiple comparison test or with a two-tailed unpaired Student's *t*-test when they were normally distributed and with Mann–Whitney test when this was not the case (GraphPad Prism Software 7.0, La Jolla, CA, USA).

## Supplementary Material



10.1242/joces.261893_sup1 Supplementary information
