# Supplementary material for: Reduced vacuolar ATPase protects mice from Friend virus infection – an unintended but instructive effect in Hif-2afl mice
Source: J Cell Sci. 2024 Jun 28;137(12):jcs261893. doi: 10.1242/jcs.261893 (PMC11234382; doi:10.1242/jcs.261893)
Supplement: Supplementary information [file joces-137-261893-s1.pdf]

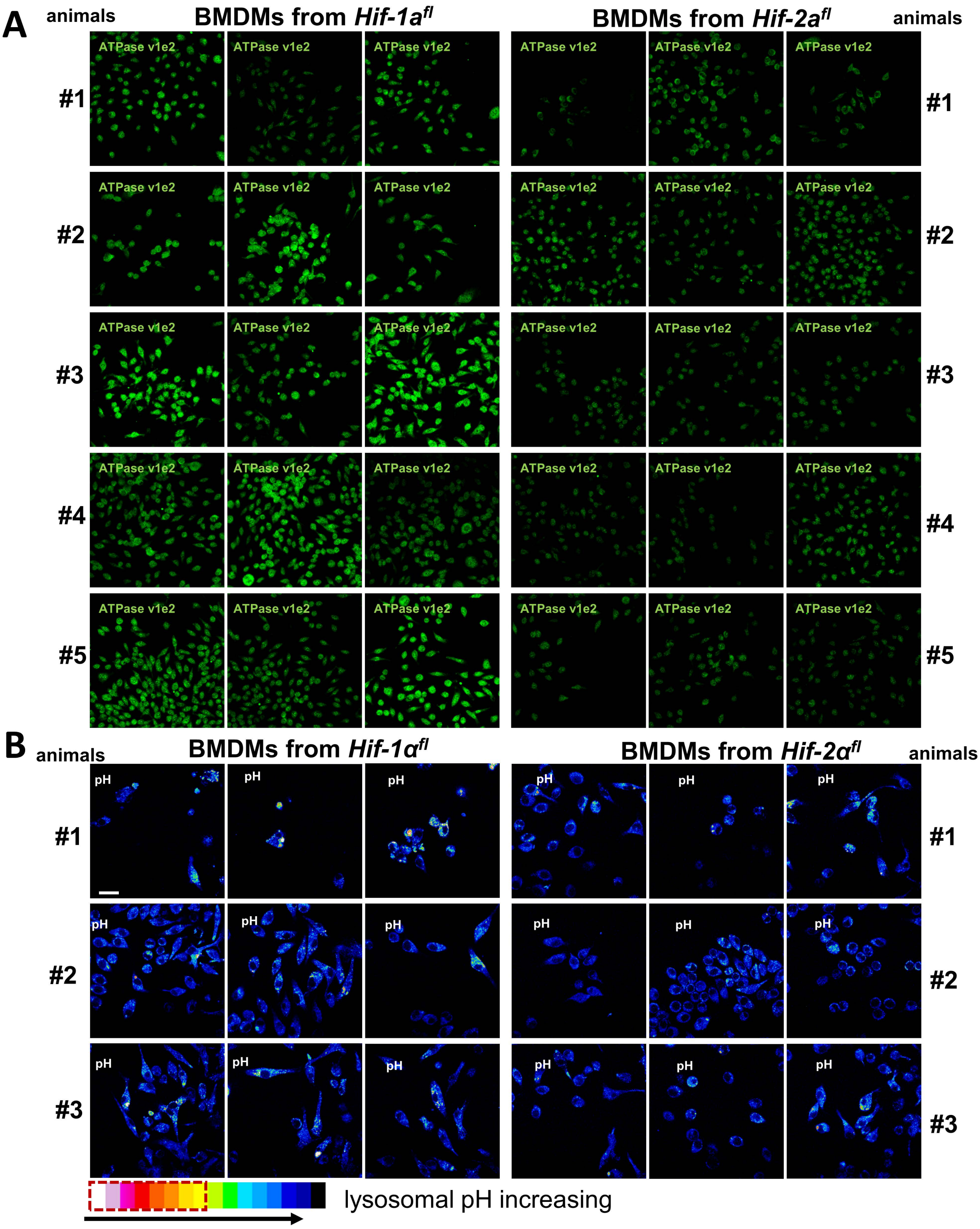

Journal of Cell Science • Supplementary information

Fig. S1. BMDMs from *Hif-2a^{fl}* mice show a reduced expression of the V-ATPase subunit E2 and show a reduced acidification of lysosomes. Representative images of all BMDMs from all analyzed individuals leading to the analysis shown in Figure 5 (B, D). (**A**):The V-ATPase subunit E2 was immunocytochemically stained in BMDMs from *Hif-1a^{fl}* and *Hif-2a^{fl}* mice: 3 representative images of 5 independent mice with floxed *Hif1a* or *Hif2a* were analyzed; scale bar: 40 µm. (**B**): The lysosomal pH has been examined with LysoSensor™ with increasing yellow to white coloring (see red dotted box) indicating a lower lysosomal pH. 3 representative images of each 3 independent mice with floxed *Hif1a* and *Hif2a* were analyzed; scale bar: 20 µm.

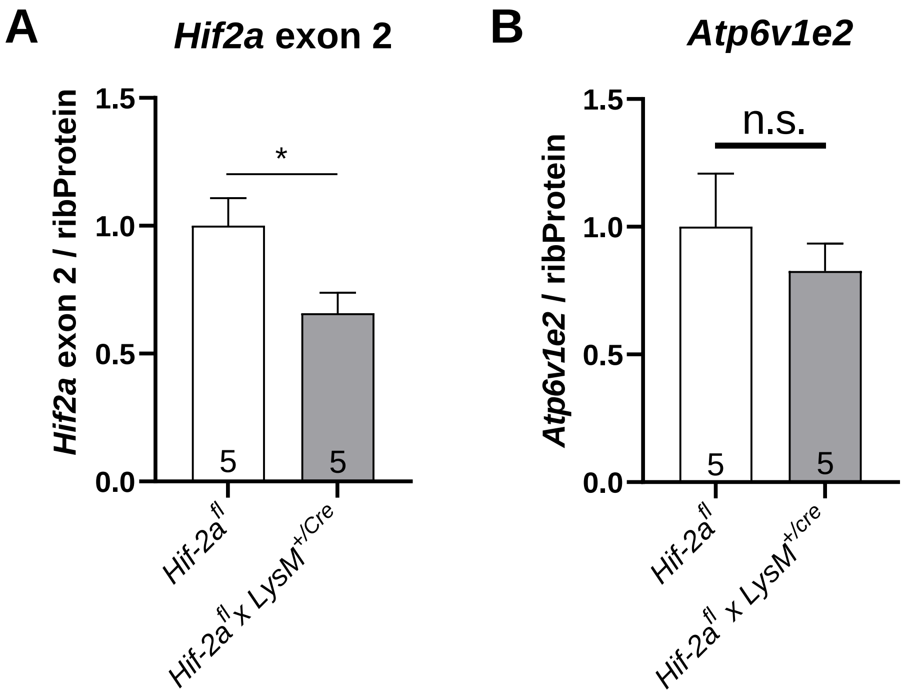

Fig. S2. Knockout of *Hif2a* does not affect the expression of *Atp6v1e2*.
To determine whether loss of functional HIF-2α alters *Atp6v1e2* expression, we performed mRNA expression analyses of BMDMs from *Hif-2a^{fl}* and *Hif-2a^{fl} x LysM$^{+/cre}$* mice. Analysis of the expression of exon2 of the *Hif2a* mRNA confirmed a significant downregulation in the BMDM culture of *Hif-2a^{fl} x LysM$^{+/cre}$* mice (A) whereas the *Atp6v1e2* expression did not differ between the two mouse strains (p=0.4667). Data were analyzed by Student´s t test (mean ± SEM). The numbers in the graphs indicate the numbers of animals tested. Analysis was performed in triplicates for each mouse. * *p-value* < 0.05; ns = not significant.

Journal of Cell Science • Supplementary information

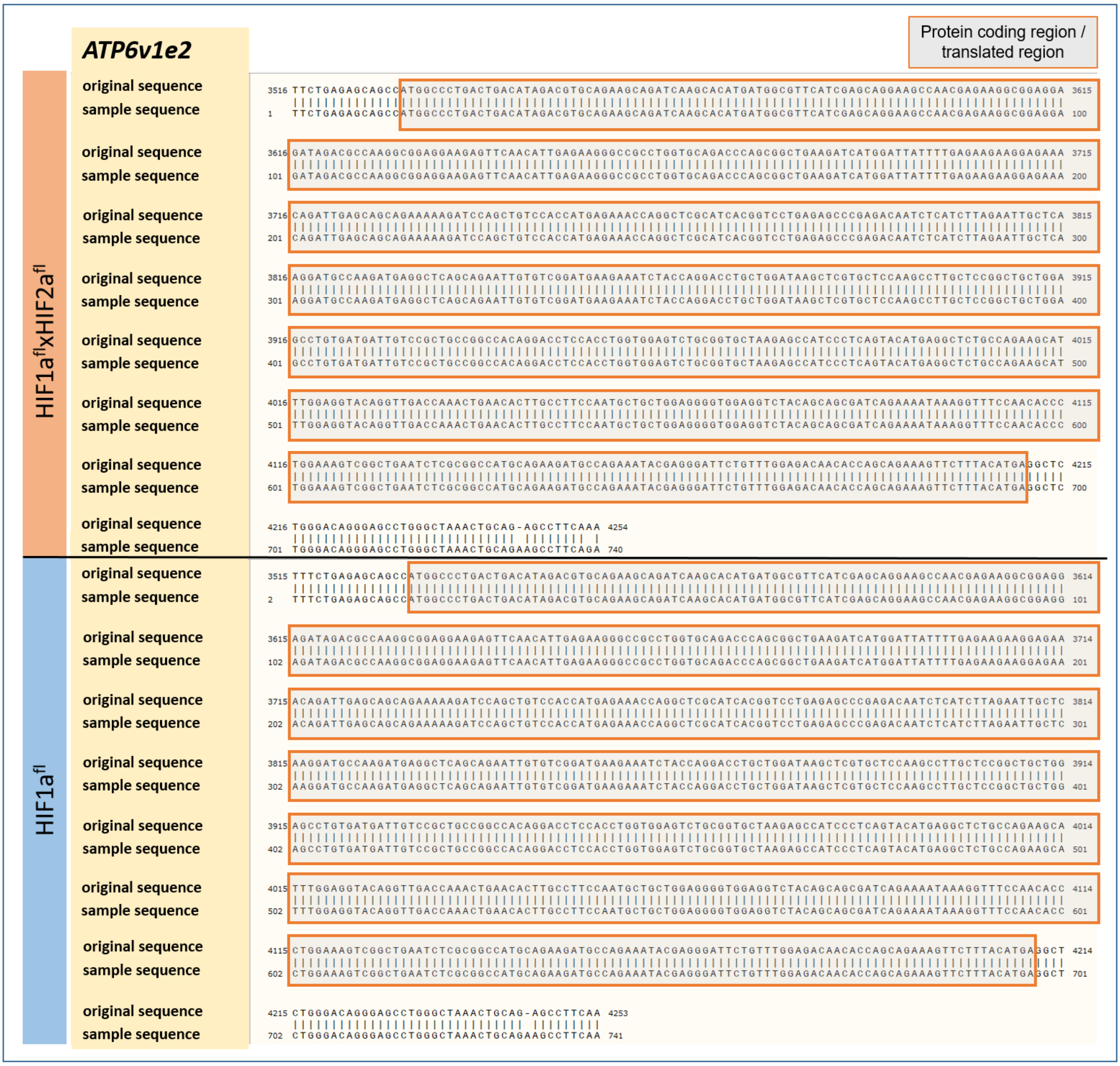

Fig. S3. The protein coding region of the *Atp6v1e2* gene is not altered in *Hif-1a^{fl}* or *Hif-1a^{fl}* x *Hif-2a^{fl}* mice.

Genomic DNA has been extracted from *Hif-1a^{fl}* and *Hif-1a^{fl}* x *Hif-2a^{fl}* mice (which behave like *Hif-2a^{fl}* with respect to FV infection, see Figure 1B) and the complete protein coding sequence of the *Atp6v1e2* gene has been sequenced. Sequence alignment of the coding region of the *Atp6v1e2* gene revealed no differences neither in *Hif-1a^{fl}* nor in *Hif-1a^{fl}* x *Hif-2a^{fl}* mice compared to the original sequence (n=3 for both mouse strains).

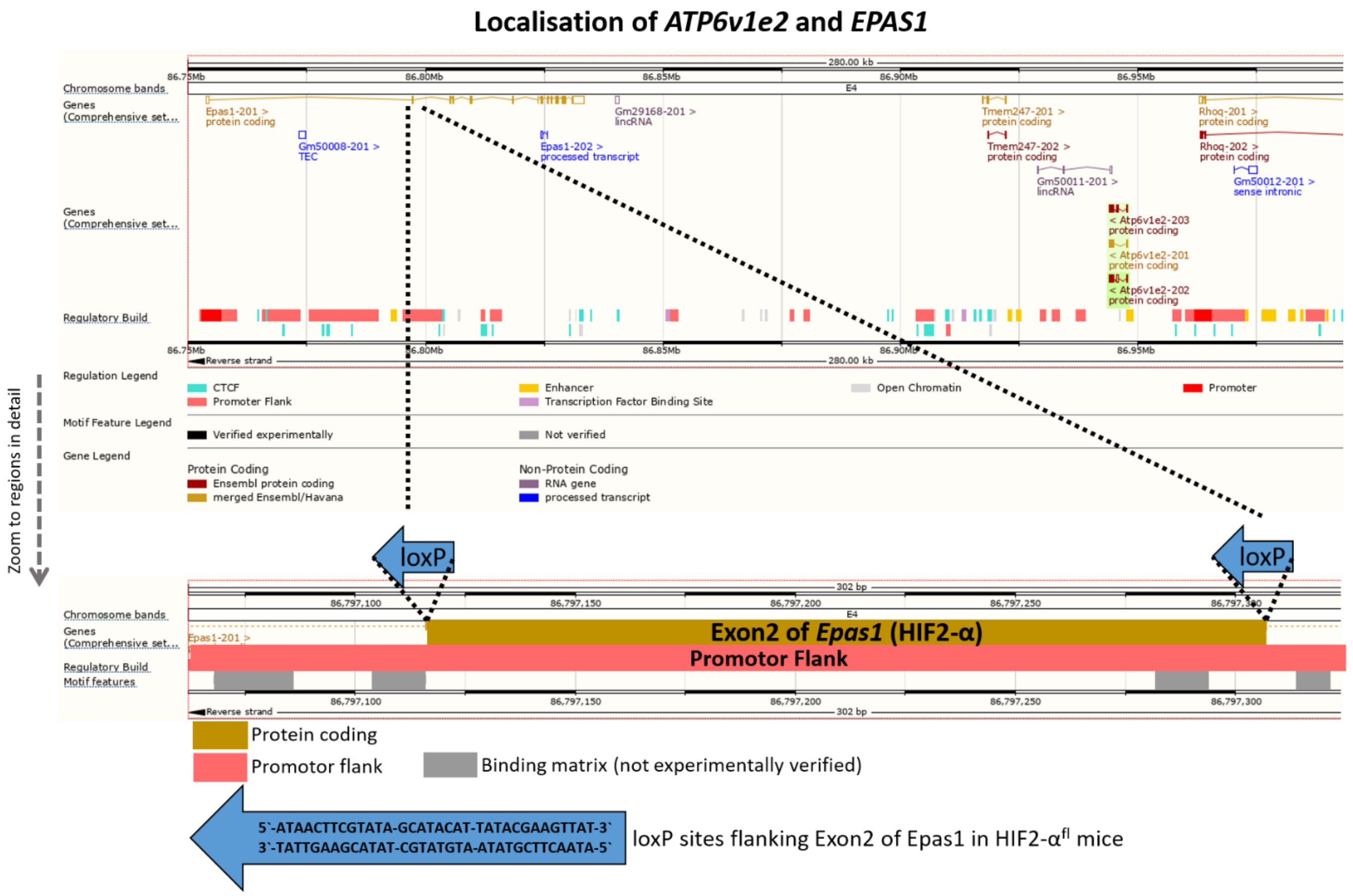

Fig. S4. Localization of the *Hif2a* and the *Atp6v1e2* gene. *Hif2a* and *Atp6v1e2* localize on chromosome 17 of the mouse. Although the loxP sites of the *Hif-2a*^{fl} mice locate in a region that is referred to as promotor flank region it is unlikely to affect the expression of the *Atp6v1e2* gene directly as the distance to the transcription start site of this gene is about 147 kilo bases. Nonetheless, the non-translated regions around exon 2 might carry indirect roles e.g. as an enhancer of transcription. The figure has been adapted from http://www.ensembl.org/ Mus_musculus/Location/View?db=core;fdb=funcgen;g=ENSMUSG00000053375; r=17:86797062- 86797363;rf=ENSMUSR00000128957;t=ENSMUST00000233995.

Journal of Cell Science • Supplementary information

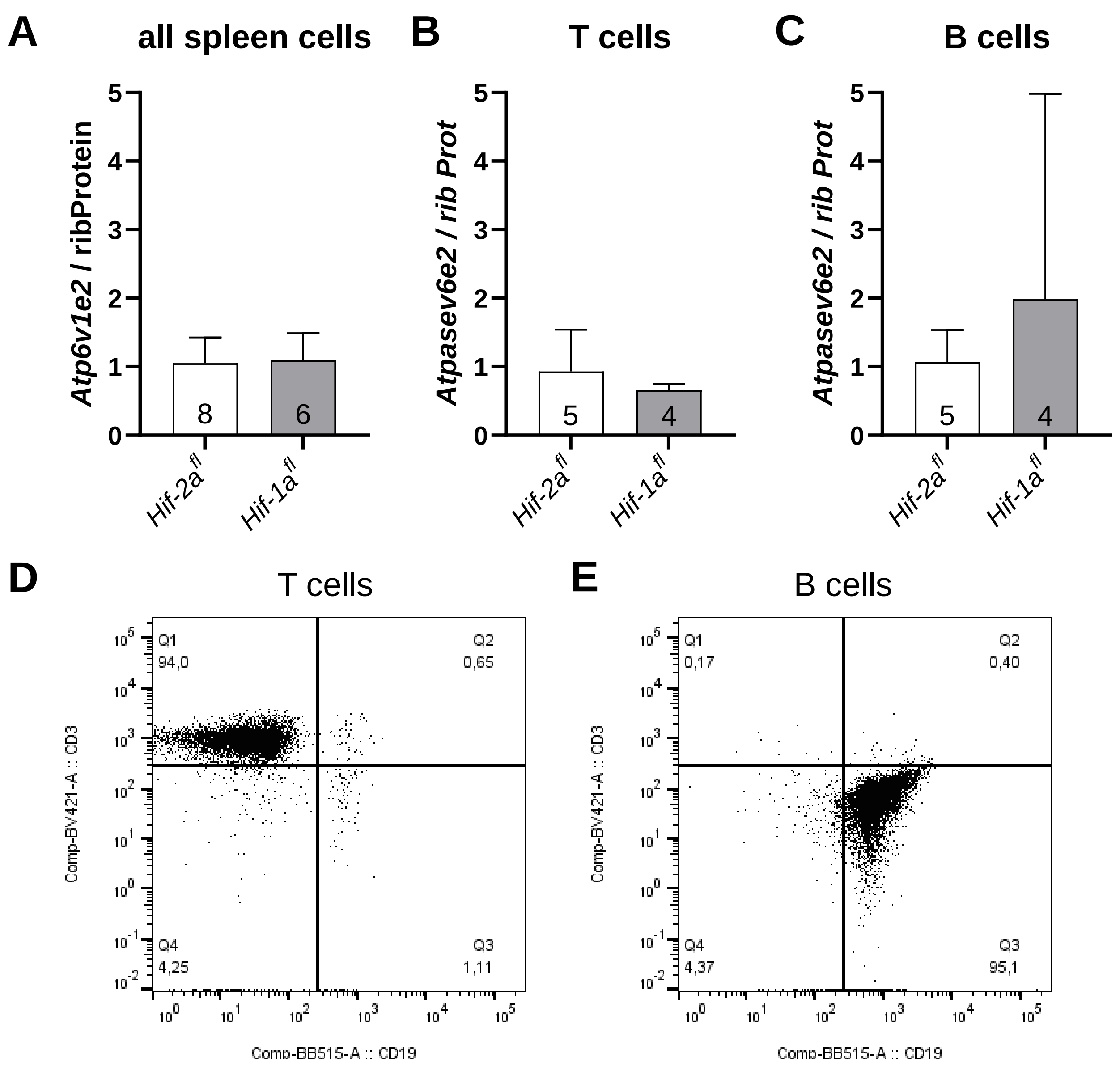

Fig. S5. The expression of *Atp6v1e2* is unaltered in total spleen cell lysates as well as in T and B cells isolated from mouse spleens.

To determine whether mRNA expression of *Atp6v1e2* is changed in other cells than in macrophages, we performed mRNA expression analyses of (A) all cells of mouse spleens, (B) MACS-enriched T cells and (C) MACS-enriched B cells from *Hif-2a*^{fl} and *Hif-1a*^{fl} mice. Analysis of the expression of the *Atp6v1e2*mRNA confirmed no differences between the two groups (mean ± SD). The numbers in the graphs indicate the numbers of animals tested. Analysis was performed in triplicates for each mouse. The purity of the enriched T and B cells was analyzed via flow cytometry and reached >90%; shown are representative dot plots (D, E).

Journal of Cell Science • Supplementary information

Table S1. Mouse strain labeling and characterization

Mouse strain labeling	Phenotype	Knockout
$Hif\text{-}1a^{fl}$	wild type	-
$Hif\text{-}1a^{fl} \times LysM^{+/cre}$	knockout	myeloid
$Hif\text{-}2a^{fl}$	wild type	-
$Hif\text{-}2a^{fl} \times LysM^{+/+}$	wild type	-
$Hif\text{-}2a^{fl} \times LysM^{+/cre}$	knockout	myeloid
$Hif\text{-}2a^{fl} \times CD11c^{+/cre}$	knockout	dendritic
$Hif\text{-}1a^{fl} \times Hif\text{-}2a^{fl}$	wild type	-
$FVB: Hif\text{-}1a^{fl}$	wild type	-

Journal of Cell Science • Supplementary information
