# Supplementary material for: Reduced vacuolar ATPase protects mice from Friend virus infection – an unintended but instructive effect in Hif-2afl mice
Source: J Cell Sci. 2024 Jun 28;137(12):jcs261893. doi: 10.1242/jcs.261893 (PMC11234382; doi:10.1242/jcs.261893)
Supplement: Supplementary file 1 [file joces-137-261893_review_history.pdf]

Reduced vacuolar ATPase protects mice from Friend virus infection - an unintended but instructive effect in *Hif-2a^{fl}* mice

Timm Schreiber, Nora Koll, Claudia Padberg, Buena de los Reyes, Theresa Quinting, Anna Malyshkina, Eric Metzen, Kathrin Sutter, Joachim Fandrey and Sandra Winning
DOI: 10.1242/jcs.261893

Editor: Kathleen Green

Review timeline
Original submission: 11 December 2023
Editorial decision: 9 January 2024
First revision received: 26 March 2024
Editorial decision: 22 April 2024
Second revision received: 23 April 2024
Accepted: 20 May 2024

Original submission

First decision letter

MS ID#: JOCES/2023/261893

MS TITLE: Floxed Hif2a mice are protected from Friend retrovirus infection, which is caused by altered vacuolar ATPase activity

AUTHORS: Timm Schreiber, Nora Koll, Claudia Padberg, Buena Delos-Reyes, Theresa Quinting, Eric Metzen, Kathrin Sutter, Joachim Fandrey, and Sandra Winning

We have now reached a decision on the above manuscript.

To see the reviewers' reports and a copy of this decision letter, please go to: https://submit-jcs.biologists.org and click on the 'Manuscripts with Decisions' queue in the Author Area. (Corresponding author only has access to reviews.)

As you will see, the reviewers raise a number of substantial criticisms that prevent me from accepting the paper at this stage. They suggest, however, that a revised version might prove acceptable, if you can address their concerns. If you think that you can deal satisfactorily with the criticisms on revision, I would be pleased to see a revised manuscript. We would then return it to the reviewers.

Please ensure that you clearly highlight all changes made in the revised manuscript. Please avoid using 'Tracked changes' in Word files as these are lost in PDF conversion.

I should be grateful if you would also provide a point-by-point response detailing how you have dealt with the points raised by the reviewers in the 'Response to Reviewers' box. Please attend to all of the reviewers' comments. If you do not agree with any of their criticisms or suggestions please explain clearly why this is so.

Reviewer 1

© 2024. Published by The Company of Biologists under the terms of the Creative Commons Attribution License (https://creativecommons.org/licenses/by/4.0/).

Advance summary and potential significance to field

In this manuscript, Schreiber et al. aimed to analyse the function of HIF-2a in the innate immune system during Friend retrovirus infection. Interestingly, HIF-2a floxed mice with intact HIF-2a gene expression were already protected against the infection. The authors present data that the expression of the gene encoding the vacuolar H+-ATPase subunit E2 (Atp6v1e2) is reduced due to the insertion of the loxP sites within the HIF-2a encoding gene. It remains unclear how exactly this insertion affects a gene in a distance of 147 kbp, although the affected area of the HIF-2a gene may serve as enhancer region for the expression of Atp6v1e2. The finding that the insertion of loxP sites can lead to an unexpected phenotype by affecting a gene that is 147 kbp away from the insertion site will be of broad interest to the cell biology community. In addition, HIF-2a is a highly important gene/protein for the cellular response to hypoxia and the majority of the current (patho)physiological knowledge of its function has been obtained in HIF-2a knockout mice. Therefore, these results are of relevance and importance also for the hypoxia field even without a detailed understanding of the underlying mechanism, but there are open questions and comments that will need to be addressed.

Comments for the author

Major comments

- Is the expression of Atp6v1e2 in BMDMs with efficient HIF-2a knockout also reduced (i.e. in the presence of a functional Cre recombinase and with demonstrated recombination of the loxP sites)?
- p. 16, line 333-335, Figure 5 C and D, Suppl. Fig. 1B: The shown and described results are difficult to interpret/to follow. The color code describes that a decreased pH (= acidification) is highlighted in shades of blue (the darker, the lower the pH). In the results description, it is described that BMDMs from HIF-2a floxed mice show an increased pH, which should be displayed in the corresponding figure in yellow, red and/or white. However, this is not the case and also the quantification indicates a decrease in yellow/red/white pixels. Therefore, the current figure suggests an acidification in lysosomes. Please, explain and correct where necessary.
- The authors include a hypothesis in the discussion that the HIF-2a gene area in and/or around the inserted loxP sites may be utilized as enhancer for the expression of Atp6v1e2. Are there any transcription factors bound in this area based on available databases? Can one or more of the same transcription factors also be found on the promoter of the Atp6v1e2 gene within such databases?
- What is known about the physiological role of the vacuolar ATPase (i.e. phenotype of corresponding knockout mice)? Could a reduced expression of the E2 subunit also be (partly) responsible for other previously reported functions of HIF-2a? This aspect should be included in the discussion.

Minor comments

- What is known about the selectivity of Concanamycin A? Could it also affect other proteins/enzymes?
- p. 16, lines 329-330: Here the authors write that the insertion of loxP sites occurred over a distance of 147 kbp. This does not fit with later descriptions that the 147 kbp describe the distance between the loxP sites and the affected Atp6v1e2 gene.
- p. 17, lines 349-350: “The spleen weight of naïve, FV-infected and FV infection in combination with ConA treatment were analyzed 7 dpi.” This sentence is not understandable and needs some correction.
- The quantifications in Figure 5D and F are difficult to understand. It would be helpful to somehow indicate what the quantifications mean in terms of pH changes (increase/decrease or similar).
- Some figure titles and legends describe investigations in mice, which indicates analyses within mice and/or tissues. However, in the corresponding figure panels results from analyses of BMDMs are displayed, which were used ex vivo. The descriptions should be adjusted accordingly.

Reviewer 2

Advance summary and potential significance to field

© 2024. Published by The Company of Biologists under the terms of the Creative Commons Attribution License (https://creativecommons.org/licenses/by/4.0/).

The manuscript by Schreiber et al shows that genetic manipulation of the C57BL/6 mice by insertion of loxP sites flanking the exon2 region of the HIF2a gene inadvertently reduced the expression of the V-ATPase subunit E2 and thereby rendered the mice resistant to the infection by the Friend virus. As a note, I am a specialist in HIF-dependent signaling but not an expert in viral infection and am not the best reviewer to comment on the importance of this finding. Whilst possibly not groundbreaking, it is important to publish unwanted/unexpected effects of genetic manipulation of mice, and here the authors provide a mechanism for this induced resistance, which is of interest for the broader community.

Comments for the author

The study is well conducted, the data provided seem robust and convincing and the experimental design is sound. My main reservation is on the title and in general, the way the story is packaged in the introduction.

I fully understand that the initial aim of the authors was to study the role of HIF2alpha in FV infection but in the end, they couldn't because of the genetic manipulation of the mice which made them resistant specifically to FV infection. They then tried to understand the reasons and found that this was due to the downregulation of the V-ATPase and lack of lysosomal acidification/function. This is the most important finding and the HIF2a function is therefore less relevant here. Whilst reading the title and the introduction, this message was not clear to me. I would suggest changing the title to reflect better the findings of the paper towards something like: Floxed HIF2a mice inadvertently altered V-ATPase expression, thereby triggering resistance to Friend virus infection. In the introduction, I would shorten the part about HIF function and eventually increase the sections about FV infection and the importance of lysosomal acidification. I found the discussion very interesting but wondered what could be the other consequences/phenotype for these mice with reduced lysosomal acidification, beyond FV infection. Surely some other functions are likely to be altered if the lysosomal pathway is affected.

Minor comments:
* The summary sentence at the beginning of the discussion (line 367-370) is very clear, and it would have been nice to have such a statement at the end of the abstract.
* The sentence line 377-379 is not correct. "not" should be moved between "is "
and "responsible"
* line 455, remove e.g.

<u>Reviewer 3</u>

Advance summary and potential significance to field

In this study, Schreiber et al. claim that a mouse line including 2 LoxP sites flanking exon 2 of HIF2 locus are protected from Friend retrovirus infection. Surprisingly this is manifested in the absence of Cre expression and therefore loss of exon 2 upon Cre-dependent recombination. Authors show evidence that the insertion of loxP sites flanking exon 2 of the Hif2a results in a decrease in the expression of the V-ATPase subunit E2 (Atp6v1e2), required for endosomal acidification and virus entry. Authors need (i) to explore a bit further the role of Atp6v1e2 expression in Friend retrovirus infection, (ii) clarify about the control mice, which is relevant to support their conclusions and (iii) consider that the title does not reflect the main finding of this study.

Comments for the author

1.- Authors mention in page 13 ‘At 7 dpi, the spleen weight of FV-infected Hif-1afll mice had nearly doubled compared to healthy control mice (Fig. 1A)’. Which is the group of mice in Figure 1A that authors consider control mice?. They should be C57BL6 control littermates mice without any LoxP site and Cre expression.

© 2024. Published by The Company of Biologists under the terms of the Creative Commons Attribution License (https://creativecommons.org/licenses/by/4.0/).

2.- Why authors use C57BL6 mice, which is a mouse strain resistant to Friend retrovirus infection showing moderate splenomegaly during acute infection as mentioned in the manuscript? It is important to show the signs of infection in control C57BL6 mice (without any LoxP site and Cre expression) in order to be compared with HIF2floxed mice and visualize better the protection claimed by the authors?

3.- According to Figure 1A and B, mice harboring two loxP sites flanking exon 2 of HIF1 locus (HIF1floxed mice) are more vulnerable to Friend retrovirus infection and therefore making C57BL6 more vulnerable to this virus?. What is the expression of Atp6v1e2 in HIF1floxed mice compared not only to HIF2floxed mice (as in Figure 5B) but also to control C57BL6 control littermates without any LoxP site and Cre expression.

4.- Authors assess the expression of Atp6v1e2 and Atp6d1in BMDMs. Because authors conclude that Atp6v1e2 is involved in the virus entry in cells vulnerable to Friend retrovirus infection, authors should assess their expression in B and T cells, correct?.

5. In Figure 1C, authors should include data of HIF1floxed/HIF2 floxed (without Cre) included in 1B. Moreover - in line with a previous comment - authors should include data from C57BL6 control littermates without any LoxP site and Cre expression in all figures.

7.- This study suggests that endocytic pathway and Atp6v1e2 expression is relevant for Friend retrovirus infection. This seem to be main conclusion and novelty of this study and this might be further investigated.

Therefore, authors should try to approach this not only using Concanamycin A (ConA) but also silencing or overexpressing Atp6v1e2 in their infection settings.

8.- The title is confusing because it does not reflect this main finding related to the role of the endocytic pathway and Atp6v1e2 expression in Friend retrovirus infection. Moreover, it includes the word ‘HIF2a’, which is also confusing because HIF2 expression is not related to the protection from this retrovirus.

First revision

Author response to reviewers' comments

Point-to-point response to the reviewers:

Reviewer 1 Advance Summary and Potential Significance to Field:
In this manuscript, Schreiber et al. aimed to analyse the function of HIF-2a in the innate immune system during Friend retrovirus infection. Interestingly, HIF-2a floxed mice with intact HIF-2a gene expression were already protected against the infection. The authors present data that the expression of the gene encoding the vacuolar H+-ATPase subunit E2 (Atp6v1e2) is reduced due to the insertion of the loxP sites within the HIF-2a encoding gene. It remains unclear how exactly this insertion affects a gene in a distance of 147 kbp, although the affected area of the HIF-2a gene may serve as enhancer region for the expression of Atp6v1e2. The finding that the insertion of loxP sites can lead to an unexpected phenotype by affecting a gene that is 147 kbp away from the insertion site will be of broad interest to the cell biology community. In addition, HIF-2a is a highly important gene/protein for the cellular response to hypoxia and the majority of the current (patho)physiological knowledge of its function has been obtained in HIF-2a knockout mice. Therefore, these results are of relevance and importance also for the hypoxia field even without a detailed understanding of the underlying mechanism, but there are open questions and comments that will need to be addressed.

We would like to thank the reviewer for his appreciation of our work and the detailed and clear advice to strengthen the outline of our manuscript.

© 2024. Published by The Company of Biologists under the terms of the Creative Commons Attribution License (https://creativecommons.org/licenses/by/4.0/).

Reviewer 1 Comments for the Author:

Major comments.

- Is the expression of Atp6v1e2 in BMDMs with efficient HIF-2a knockout also reduced (i.e. in the presence of a functional Cre recombinase and with demonstrated recombination of the loxP sites)?

This is an excellent question. We have compared the mRNA expression of Atp6v1e2 *in BMDMs isolated from* Hif2a^{fl} *and* Hif2a^{fl} x Lyz2-cre *mice (with verified knockout efficiency) and found them to be identical. We added these data as new supplemental figure 2 (described in ll. 209-211 in the results section) and we have included this point in the discussion (ll. 270-274).*

- p. 16, line 333-335, Figure 5 C and D, Suppl. Fig. 1B: The shown and described results are difficult to interpret/to follow. The color code describes that a decreased pH (= acidification) is highlighted in shades of blue (the darker, the lower the pH). In the results description, it is described that BMDMs from HIF-2a floxed mice show an increased pH, which should be displayed in the corresponding figure in yellow, red and/or white. However, this is not the case and also the quantification indicates a decrease in yellow/red/white pixels. Therefore, the current figure suggests an acidification in lysosomes. Please, explain and correct where necessary.

We thank the reviewer for this comment and apologize for the confusion. We have now corrected both figures and have changed the description in the figures, which was indeed wrong. We corrected "lysosomal pH decreasing" to "lysosomal pH increasing" in both figures and have included the pH change also in the descriptions of figures 5D and 5F, respectively. We have adapted the figure legends carefully but have not made any changes in the manuscript as the text delivers the correct information.

- The authors include a hypothesis in the discussion that the HIF-2a gene area in and/or around the inserted loxP sites may be utilized as enhancer for the expression of Atp6v1e2. Are there any transcription factors bound in this area based on available databases? Can one or more of the same transcription factors also be found on the promoter of the Atp6v1e2 gene within such databases?

This is a very good suggestion. Unfortunately, there is no evidence that the enhancer found in the region of exon 2 is associated with the Atp6v1e2. We included the following statement in the discussion (ll. 359-365):
A known enhancer (ID: ENSMUSR00000128964) lies on chromosome 17 in the region of 87.100.202 - 87.111.468 bp, overlapping with exon 2 of Hif2a (Fergal et al. Nucleic Acids Res. 2023, 51(D1):D933-D941). This enhancer region is active in the adult murine spleen and is associated with different proteins (e.g. histone H3 and the CCCTC-binding factor CTCF) that regulate the 3D structure of chromatin and therefore, gene regulation. However, to our knowledge there is no evidence that this enhancer is associated with the expression of Atp6v1e2 so far.

- What is known about the physiological role of the vacuolar ATPase (i.e. phenotype of corresponding knockout mice)? Could a reduced expression of the E2 subunit also be (partly) responsible for other previously reported functions of HIF-2a? This aspect should be included in the discussion.

This is interesting. The v-ATPase is a very important protein in nearly every cell of the body, including the brain, lung, bone, skin, kidney and even the ear and nose. Consequently, a global knock out of the v-ATPase leads to early embryonic lethality. Due to the wide variety of functions, it is difficult to rule out the interference of the insertion of the loxP sites with the function of the v-ATPase in any *findings produced with this mouse strain. This is why we think our findings are of importance for the whole scientific community that works with Hif-2a^{fl} mice. We included the following statement in the manuscript (ll. 397-407):*
If the interference of the inserted loxP sites with the function of the V-ATPase is responsible for other findings whilst using this particular mouse strain will be hard to interpret, as the V-ATPase plays an important role in a variety of different biological processes, like toxin delivery, membrane targeting, apoptosis, regulation of cytoplasmic pH, proteolytic process, acidification of intracellular systems, autophagy, and many more (Eaton et al. 2021; Kenney and Benarroch 2015). In addition, unspecific effects due to the insertion of the loxP sites alone will be immediately unraveled as they would also manifest in Cre-negative siblings. This corroborates the need for suitable control animals in animal studies. To the best of our knowledge, this strategy has excluded so far that the reduced expression of V-ATPase might be responsible for

© 2024. Published by The Company of Biologists under the terms of the Creative Commons Attribution License (https://creativecommons.org/licenses/by/4.0/).

findings that have since then been alluded to HIF-2a deficiency.

Minor comments

• What is known about the selectivity of Concanamycin A? Could it also affect other proteins/enzymes?

Concanamycin A is a macrolide antibiotic and (due to its complex chemical structure) a highly specific inhibitor of the v-ATPase, which we have used at very low concentrations (in vivo: 12 ng ConA/g mouse weight). To our knowledge there are no other mammalian targets described in the literature. Painter et al. ([1]doi: 10.1073/pnas.2008615117*) have described a very potent interaction with the Nef protein of HIV, which impedes the immune evasion of HIV. As Nef is a protein which is restricted to human and simian viruses (*[2]*doi: 10.1016/j.mam.2010.05.003), this should not be of relevance for FV infection. We have changed the manuscript accordingly (ll. 235-238):*
However, inhibition of the V-ATPase using the highly specific inhibitor Concanamycin A (ConA) at a nanomolar concentration during FV infection in spleenoids cultured from Hif-1a^{fl} mice resulted in reduced Ter119^{+} erythroblast proliferation and viral replication (Fig. 6B,C).

• p. 16, lines 329-330: Here the authors write that the insertion of loxP sites occurred over a distance of 147 kbp. This does not fit with later descriptions that the 147 kbp describe the distance between the loxP sites and the affected Atp6v1e2 gene.
We thank the reviewer for this comment, which is absolutely correct. We excuse for the inaccuracy and have changed the text (ll. 216-220) as follows:
The insertion of loxP sites flanking exon 2 of the Hif2a gene occurred approximately 147 kilobase pairs (kbp) away from the Atp6v1e2 gene (see suppl. Fig. 4) but nevertheless caused a suppression with functional consequences in macrophages.

• p. 17, lines 349-350: “The spleen weight of naïve, FV-infected and FV infection in combination with ConA treatment were analyzed 7 dpi.” This sentence is not understandable and needs some correction.
We have corrected the respective sentence to (ll. 241-243):
The spleen weight of naïve and FV-infected mice as well as those of mice that were treated with ConA before and whilst FV infection were analyzed 7 dpi.

• The quantifications in Figure 5D and F are difficult to understand. It would be helpful to somehow indicate what the quantifications mean in terms of pH changes (increase/decrease or similar).
We thank the reviewer for this excellent suggestion and have adapted the respective figures 5D and 5F as described in our comment above.

• Some figure titles and legends describe investigations in mice, which indicates analyses within mice and/or tissues. However, in the corresponding figure panels results from analyses of BMDMs are displayed, which were used ex vivo. The descriptions should be adjusted accordingly.
We would like to thank the reviewer for this comment. We have carefully reviewed all figures and figure legends and have corrected this in figure 5 and in the supplementary figure 1.

*****

Reviewer 2 Advance Summary and Potential Significance to Field:
The manuscript by Schreiber et al shows that genetic manipulation of the C57BL/6 mice by insertion of loxP sites flanking the exon2 region of the HIF2a gene inadvertently reduced the expression of the V-ATPase subunit E2 and thereby rendered the mice resistant to the infection by the Friend virus. As a note, I am a specialist in HIF-dependent signaling but not an expert in viral infection and am not the best reviewer to comment on the importance of this finding. Whilst possibly not groundbreaking, it is important to publish unwanted/unexpected effects of genetic manipulation of mice, and here the authors provide a mechanism for this induced resistance, which is of interest for the broader community.

Reviewer 2 Comments for the Author:

© 2024. Published by The Company of Biologists under the terms of the Creative Commons Attribution License (https://creativecommons.org/licenses/by/4.0/).

The study is well conducted, the data provided seem robust and convincing and the experimental design is sound. My main reservation is on the title and in general, the way the story is packaged in the introduction.

I fully understand that the initial aim of the authors was to study the role of HIF2alpha in FV infection, but in the end, they couldn't because of the genetic manipulation of the mice which made them resistant specifically to FV infection. They then tried to understand the reasons and found that this was due to the downregulation of the V-ATPase and lack of lysosomal acidification/function. This is the most important finding and the HIF2a function is therefore less relevant here. Whilst reading the title and the introduction, this message was not clear to me. I would suggest changing the title to reflect better the findings of the paper towards something like: Floxed HIF2a mice, inadvertently altered V-ATPase expression, thereby triggering resistance to Friend virus infection. In the introduction, I would shorten the part about HIF function and eventually increase the sections about FV infection and the importance of lysosomal acidification.
We would like to thank the reviewer for his appreciation of our work and the recommendations for improving our manuscript. We have taken these into account and have therefore changed the title of the manuscript to:

Inhibition of vacuolar ATPase protects mice from Friend virus infection - a lesson learned from an unintended side effect in Hif-2a^{fl} *mice*.

In addition, we have carefully revised the introduction and have shortened the HIF section but expanded especially the part about FV infection.

I found the discussion very interesting, but wondered what could be the other consequences/phenotype for these mice with reduced lysosomal acidification, beyond FV infection. Surely some other functions are likely to be altered if the lysosomal pathway is affected.
This is an excellent point, thus hard to answer without speculating too much. We have included this in the discussion section (ll. 397-402) but have consciously tried to avoid overinterpretation here as we have not checked any of these points experimentally.

Minor comments:
* The summary sentence at the beginning of the discussion (line 367-370) is very clear, and it would have been nice to have such a statement at the end of the abstract.
We thank the reviewer for this comment and have added a summarizing sentence about murine FV susceptibility at a suitable position of the abstract (ll. 32-34).
* The sentence line 377-379 is not correct. "not" should be moved between "is " and "responsible"
Thank you for this comment, we have corrected the respective sentence. It can now be found in ll. 271/272:
We conclude that ~~not~~ myeloid knockout of HIF-2a is not responsible for this effect [...].

line 455, remove e.g.
We have removed the abbreviation (now l. 359):
The region around Hif2a exon 2 might ~~e.g.,~~ function as an enhancer of Atp6v1e2 transcription.
*****
Reviewer 3 Advance Summary and Potential Significance to Field:
In this study, Schreiber et al. claim that a mouse line including 2 LoxP sites flanking exon 2 of HIF2 locus are protected from Friend retrovirus infection. Surprisingly this is manifested in the absence of Cre expression and therefore loss of exon 2 upon Cre-dependent recombination. Authors show evidence that the insertion of loxP sites flanking exon 2 of the Hif2a results in a decrease in the expression of the V-ATPase subunit E2 (Atp6v1e2), required for endosomal acidification and virus entry. Authors need (i) to explore a bit further the role of Atp6v1e2 expression in Friend retrovirus infection, (ii) clarify about the control mice, which is relevant to support their conclusions and (iii) consider that the title does not reflect the main finding of this study.

We would like to thank the reviewer for a careful, detailed, and well-balanced examination of our work and the suggestions to amend our manuscript.

© 2024. Published by The Company of Biologists under the terms of the Creative Commons Attribution License (https://creativecommons.org/licenses/by/4.0/).

Reviewer 3 Comments for the Author:
1.- Authors mention in page 13 'At 7 dpi, the spleen weight of FV-infected Hif-1afll mice had nearly doubled compared to healthy control mice (Fig. 1A)'. Which is the group of mice in Figure 1A that authors consider control mice?. They should be C57BL6 control littermates mice without any LoxP site and Cre expression.

We thank the reviewer for this comment and excuse for not being accurate here. The mice we used in this study were purchased from the Jax laboratory (please refer to the M&M section for the respective stock numbers). The mice have originally been produced on a mixed background and were then cross-bred to C57BL/6 mice; they are homozygously floxed (we have added this fact explicitly in l. 124 now) and heterozygously express Cre recombinase after crossbreeding with the given Cre-mice. Thus, the breeding scheme will exclude C57BL/6 wildtype mice as littermate controls. Whenever we refer to "control mice" in our manuscript, we therefore mean Hif-1a^{fl} or Hif-2a^{fl} mice without Cre expression. These are the littermate, knockout-free controls but still remain genetically modified mice.

2.- Why authors use C57BL6 mice, which is a mouse strain resistant to Friend retrovirus infection showing moderate splenomegaly during acute infection as mentioned in the manuscript? It is important to show the signs of infection in control C57BL6 mice (without any LoxP site and Cre expression) in order to be compared with HIF2floxed mice and visualize better the protection claimed by the authors?

We thank the reviewer for raising this understandable concern and would like to explain our thoughts in more detail here. The study presented in this manuscript has been designed in addition to the findings we obtained before, namely that myeloid HIF-1 is important in FV infection ([3]Schreiber et al., 2017). These data have been evaluated in mice carrying a C57BL/6 background. To provide the best comparability, we used mice with the same genetic background for this project.
As said above, it is unfortunately not possible to breed littermate controls with a "real wildtype" C57BL/6 background, which excludes C57BL/6 mice as adequate controls for our manuscript. Nonetheless, we are happy to provide data to the reviewer from C57BL/6 mice that have been infected within our animal housing, and with the same lot and amounts of FV compared to our animals. These data show a comparable viral load in the spleens of C57BL/6 mice seven days after infection (two independent experiments with 7 and 8 animals, respectively) to those we have found in the Hif-1a^{fl} *mice and fully support the protection of* Hif-2a^{fl} *mice against FV infection:*

NOTE: We have removed unpublished data that had been provided for the referees in confidence.

We have emphasized this fact in ll. 284/285 of the manuscript now.

3.- According to Figure 1A and B, mice harboring two loxP sites flanking exon 2 of HIF1 locus (HIF1floxed mice) are more vulnerable to Friend retrovirus infection and therefore making C57BL6 more vulnerable to this virus?. What is the expression of Atp6v1e2 in HIF1floxed mice compared not only to HIF2floxed mice (as in Figure 5B) but also to control C57BL6 control littermates without any LoxP site and Cre expression.
We thank the reviewer for these plausible questions. As we presented above, Hif-1a^{fl} *mice behave as C57BL/6 wildtype mice with respect to the infectibility by FV. Regarding the expression analysis of Atp6v1e2 in* Hif-1a^{fl} *mice compared to C57BL/6 mice we would like to kindly refer to our argumentation above that wildtype C57BL/6 animals cannot serve as adequate control animals in our experimental setting. Therefore, we do not present additional data here.*

4.- Authors assess the expression of Atp6v1e2 and Atp6d1in BMDMs. Because authors conclude that Atp6v1e2 is involved in the virus entry in cells vulnerable to Friend retrovirus infection, authors should assess their expression in B and T cells, correct?.
We thank the reviewer for this suggestion. Recent data reveal that T cells are not considered a main target of Friend Virus and B-cells are infected later than myeloid cells (Windmann et al., mBio 2019). Nonetheless, T- and B-cells are the main populations in the spleen except from myeloid cells and nucleus-deficient erythrocytes. Therefore, we have isolated T- and B- cells

© 2024. Published by The Company of Biologists under the terms of the Creative Commons Attribution License (https://creativecommons.org/licenses/by/4.0/).

from the spleens of Hif-1a^{fl} *and* Hif-2a^{fl} *mice and analyzed their basal expression of the* Atp6v1e2 *mRNA (see new Suppl. Fig 5). However, as described in ll. 220/221 we found no difference between* Hif-2a^{fl} *and* Hif-1a^{fl} *mice (serving as "infectable controls" of a comparable genetic background here). We have discussed this as follows (ll. 368-386):*

Interestingly, T and B cells of the spleens of Hif-2a^{fl} mice did not show a reduced expression of the Atp6v1e2 *mRNA (Suppl. Fig.5). There are some possible explanations for this finding. First, it is not known if the enhancer, located in exon 2, is active in lymphocytes. Second, the V1 domain of the ATPase contains tissue-specific subunit isoforms including B, C, E, and G. Therefore, the E2 subunit* Atp6v1e2 *encodes for might not be of importance in lymphocytes. And third, the expression of* Hif2a *is remarkably lower in B- and T- cells compared to BMDMs, which might influence the expression of the Atp6v1e2 (delta Ct = Ct(*Hif2a*)-Ct(*Rps16*): BMDMs: 10.74 ± 0.81; B cells: 12.13 ± 1.16; T cells: 13.44 ± 1.34; mean ± SD of 10 mice per group; data not shown). All in all,* Hif-2a^{fl} *mice do not show any signs of FV infection, although there is no change in the expression of* Atp6v1e2 *in other cells than macrophages. This might be due to the fact that the injected viral titers were too low to infect lymphocytes without prior replication in macrophages. Honke et al. have already described an enforced viral replication by macrophages in murine vesicular stomatitis virus (VSV) and lymphocytic choriomeningitis virus (LCMV) infection; a mechanism that might be worth investigating during FV infection as well (Honke et al. 2011). Of note, T cells are not considered the main target of FV, and B cells have been shown to be infected at later time points compared to myeloid cells (Windmann et al. 2019), which points to an outstanding role for the vacuolar ATPase in macrophages during FV spread.*

5.- In Figure 1C, authors should include data of HIF1floxed/HIF2 floxed (without Cre) included in 1B. Moreover - in line with a previous comment - authors should include data from C57BL6 control littermates without any LoxP site and Cre expression in all figures.
We thank the reviewer for this remark. In line with the comments above, we think that we have chosen the most adequate controls possible for our experiments and therefore do not present additional data here.

7.- This study suggests that endocytic pathway and Atp6v1e2 expression is relevant for Friend retrovirus infection. This seem to be main conclusion and novelty of this study and this might be further investigated. Therefore, authors should try to approach this not only using Concanamycin A (ConA) but also silencing or overexpressing Atp6v1e2 in their infection settings.
Thank you very much for this interesting suggestion. Indeed, this would largely improve the understanding of the role of the Atp6v1e2 *in infections. Unfortunately, FV infections cannot be studied in cell culture. That is why we developed the spleenoid culture for our experiments, where we were able to investigate some aspects of the infection. However, as spleenoids are a primary mixed cell culture, they are not suitable for genetic modifications. Even in a highly efficient CRISPR/CAS setting, these mixed cultures cannot be transfected sufficiently. Each cell type would have a different transfection efficiency and a clonal expansion of transfected cells would not be possible, because of their limited or absent proliferation capacity. Another option are genetically modified mouse strains. Mice harboring a knockout/overexpression of the* Atp6v1e2 *gene are currently not available and we would have to breed our own mouse strain. Under German law, this would require a proposal for animal testing, breeding of the new strain and a critical characterization of the mice regarding animal suffering before the actual experiments could be performed. And although these experiments would improve our findings remarkably, we are regrettably not able to perform these experiments in a time frame consistent with a timely revision of our manuscript.*

8.- The title is confusing because it does not reflect this main finding related to the role of the endocytic pathway and Atp6v1e2 expression in Friend retrovirus infection. Moreover, it includes the word 'HIF2a', which is also confusing because HIF2 expression is not related to the protection from this retrovirus.
We thank the reviewer for this absolutely comprehensible comment. We have now changed the title to
Inhibition of vacuolar ATPase protects mice from Friend virus infection - a lesson learned from an unintended side effect in Hif-2a^{fl} mice to make this clearer.

References:

© 2024. Published by The Company of Biologists under the terms of the Creative Commons Attribution License (https://creativecommons.org/licenses/by/4.0/).

1) Mark M. Painter, Gretchen E. Zimmerman, Madeline S. Merlino, Andrew W. Robertson, Valeri H. Terry, Xuefeng Ren, Megan R. McLeod, Lyanne Gomez-Rodriguez, Kirsten A. Garcia, Jolie A. Leonard, Kay E. Leopold, Andrew J. Neevel, Jay Lubow, Eli Olson, Alicja Piechocka- Trocha, David R. Collins, Ashootosh Tripathi, Malini Raghavan, Bruce D. Walker, James H. Hurley, David H. Sherman, and Kathleen L. Collins. Concanamycin A counteracts HIV-1 Nef to enhance immune clearance of infected primary cells by cytotoxic T lymphocytes. PNAS, 117 (38) 23835-23846. https://doi.org/10.1073/pnas.2008615117.
2) Laguette N, Brégnard C, Benichou S, Basmaciogullari S. Human immunodeficiency virus (HIV) type-1, HIV-2 and simian immunodeficiency virus Nef proteins. Mol Aspects Med. 2010 Oct;31(5):418-33. doi: 10.1016/j.mam.2010.05.003 . Epub 2010 Jun 4. PMID: 20594957 .
3) Schreiber T, Quinting T, Dittmer U, Fandrey J, Sutter K. Hypoxia-inducible factor 1α is Essential for Macrophage-mediated Erythroblast Proliferation in Acute Friend Retrovirus Infection. Sci Rep. 2017 Dec 8;7(1):17236. doi: 10.1038/s41598-017-17324-y.

Second decision letter

MS ID#: JOCES/2023/261893

MS TITLE: Reduced vacuolar ATPase protects mice from Friend virus infection - an unintended but instructive effect in *Hif-2a*^{fl} mice

AUTHORS: Timm Schreiber, Nora Koll, Claudia Padberg, Buena Delos Reyes, Theresa Quinting, Anna Malyshkina, Eric Metzen, Kathrin Sutter, Joachim Fandrey, and Sandra Winning

We have now reached a decision on the above manuscript.

To see the reviewers' reports and a copy of this decision letter, please go to: https://submit-jcs.biologists.org and click on the 'Manuscripts with Decisions' queue in the Author Area. (Corresponding author only has access to reviews.)

As you will see, the reviewers gave favourable reports but raised some critical points that will require amendments to your manuscript. I hope that you will be able to carry these out because I would like to be able to accept your paper, depending on further comments from reviewers.

Please ensure that you clearly highlight all changes made in the revised manuscript. Please avoid using 'Tracked changes' in Word files as these are lost in PDF conversion.

I should be grateful if you would also provide a point-by-point response detailing how you have dealt with the points raised by the reviewers in the 'Response to Reviewers' box. Please attend to all of the reviewers' comments. If you do not agree with any of their criticisms or suggestions please explain clearly why this is so.

Reviewer 1

Advance summary and potential significance to field

In this manuscript, Schreiber et al. show that widely used HIF-2a floxed mice are protected against Friend retrovirus infection due to the insertion of the flox sequences within the DNA, likely disrupting an enhancer region for the expression of Atp6v1e2. Thus, these results further highlight the relevance of the vacuolar ATPase for Friend retrovirus infection. These results will therefore be relevant for the hypoxia research field and beyond.

Comments for the author

© 2024. Published by The Company of Biologists under the terms of the Creative Commons Attribution License (https://creativecommons.org/licenses/by/4.0/).

The authors have supplied sufficient data and answers to my comments and requests.

Reviewer 2

Advance summary and potential significance to field

The authors have answered my queries and have adequately modified their manuscript in the revised version

Comments for the author

NA, see above

Reviewer 3

Advance summary and potential significance to field

Authors have addressed partially my comments. Therefore authors should address the following new comments.

1.- Authors mention that ‘Whenever we refer to “control mice” in our manuscript we therefore mean Hif-1afl or Hif-2afl mice without Cre expression’. However, in Figure 1A, HIF1a fl is the control mice but not HIF2fl, correct?. In other words in Figure 1A, HIF2fl are the mice in which the phenotype is observed when compared with HIF1fl (control mice), correct?

2.- Authors mention that ‘Thus, the breeding scheme will exclude C57BL/6 wildtype mice as littermate controls’. However, HIF2LoXP/LoxP mice could be crossed first with wild type C57/BL6 mice, and the resulting heterozygous Hif2LoxP/wt mice can be crossed again to obtain wild type C57/BL6 mice, Hif2LoxP/wt and HIF2LoXP/LoxP littermates. Authors should comment about this possible breeding scheme.

3.- In this revised version of the manuscript, authors claim that Atp6v1e2 expression is not reduced upon HIF2a gene inactivation in Hif-2afl x LysM+/cre BMDMs. However, in Suppl. Figure 2, it seems that there is trend of reduced Atp6v1e2 expression (panel B) importantly in conditions in which HIF2 expression is partially reduced (panel A).
Authors should comment, why HIF2a expression is not completely reduced in Hif-2afl x LysM+/cre BMDMs?. Moreover, it is important to compare HIF2a expression in Hif-2afl mice and control C57BL/6 without LoxP sites following the breeding scheme proposed in comment #2 above); or alternatively comparing Hif-1afl or Hif-2afl mice.

4.- The new title of the manuscript reflects now better the findings shown in this study, which suggest the role of vacuolar ATPase in macrophages in Friend virus infection. However, this conclusion is based on the use of Concanamycin A (Con A), which might be discussed as a limitation of the study.

Comments for the author

Authors have addressed partially my comments. Therefore authors should address the following new comments.

1.- Authors mention that ‘Whenever we refer to “control mice” in our manuscript we therefore mean Hif-1afl or Hif-2afl mice without Cre expression’. However, in Figure 1A, HIF1a fl is the control mice but not HIF2fl, correct?. In other words in Figure 1A, HIF2fl are the mice in which the phenotype is observed when compared with HIF1fl (control mice), correct?

© 2024. Published by The Company of Biologists under the terms of the Creative Commons Attribution License (https://creativecommons.org/licenses/by/4.0/).

2.- Authors mention that ‘Thus, the breeding scheme will exclude C57BL/6 wildtype mice as littermate controls’. However, HIF2LoXP/LoxP mice could be crossed first with wild type C57/BL6 mice, and the resulting heterozygous Hif2LoxP/wt mice can be crossed again to obtain wild type C57/BL6 mice, Hif2LoxP/wt and HIF2LoXP/LoxP littermates. Authors should comment about this possible breeding scheme.

3.- In this revised version of the manuscript, authors claim that Atp6v1e2 expression is not reduced upon HIF2a gene inactivation in Hif-2afl x LysM+/cre BMDMs. However, in Suppl. Figure 2, it seems that there is trend of reduced Atp6v1e2 expression (panel B) importantly in conditions in which HIF2 expression is partially reduced (panel A).
Authors should comment, why HIF2a expression is not completely reduced in Hif-2afl x LysM+/cre BMDMs?. Moreover, it is important to compare HIF2a expression in Hif-2afl mice and control C57BL/6 without LoxP sites following the breeding scheme proposed in comment #2 above); or alternatively comparing Hif-1afl or Hif-2afl mice.

4.- The new title of the manuscript reflects now better the findings shown in this study, which suggest the role of vacuolar ATPase in macrophages in Friend virus infection. However, this conclusion is based on the use of Concanamycin A (Con A), which might be discussed as a limitation of the study.

Second revision

Author response to reviewers' comments

Point-to-point response to the reviewer

We thank the reviewer for the careful revision of our revised manuscript. We are happy to meet the new comments and have tried to address all of them properly as follows:

1.- Authors mention that ‘Whenever we refer to “control mice” in our manuscript, we therefore mean Hif-1afl or Hif-2afl mice without Cre expression’. However, in Figure 1A, HIF1a fl is the control mice but not HIF2fl, correct?. In other words, in Figure 1A, HIF2fl are the mice in which the phenotype is observed when compared with HIF1fl (control mice), correct?
This is absolutely true. We thank the reviewer for unraveling this inaccuracy in our argumentation. To make this absolutely clear to the readers, we have changed the manuscript in ll. 118-119 as follows: “At 7 dpi, the spleen weight of FV-infected *Hif-1a*^{fl} mice (serving as control mice with already described characteristics of FV infection here) had nearly doubled compared to healthy, controluninfected mice (Fig. 1A).”

2.- Authors mention that ‘Thus, the breeding scheme will exclude C57BL/6 wildtype mice as littermate controls’. However, HIF2LoXP/LoxP mice could be crossed first with wild type C57/BL6 mice, and the resulting heterozygous Hif2LoxP/wt mice can be crossed again to obtain wild type C57/BL6 mice, Hif2LoxP/wt and HIF2LoXP/LoxP littermates. Authors should comment about this possible breeding scheme.
We thank the reviewer for this comment. We would like to emphasize that we can fully understand the wish to obtain the best comparability to wildtype mice as possible. Unfortunately, we still think that there are influential reasons that prevent the use of wildtype C57/BL6 mice as littermate controls in our experimental setting.
Our mice are not only double-floxed for Hif2a, *but also carry a heterozygous Cre expression in one of the parental animals. We would therefore need to breed* Hif2a$^{fl/wt}$ *(*Cre$^{wt/wt}$*) mice with* Hif2a$^{fl/wt}$ *x* Cre$^{ki/wt}$ *mice. This would reveal 50% of heterozygously floxed Hif2a mice (3/4 expressing* Cre$^{wt/wt}$*; 1/4 expressing* Cre$^{ki/wt}$ *), which could not be used for the experiments. The remaining 50% of breeded animals would show the following genoytpes:*

© 2024. Published by The Company of Biologists under the terms of the Creative Commons Attribution License (https://creativecommons.org/licenses/by/4.0/).

C57Bl/6 (Hif2a$^{wt/wt}$ Cre$^{wt/wt}$)*: 18.75% (of total animals)*
Hif2a$^{wt/wt}$ Cre$^{ki/wt}$*: 6.25% (would also not be used for experiments)*
Hif2a$^{fl/fl}$ Cre$^{wt/wt}$*: 18.75%*
Hif2a$^{fl/fl}$ Cre$^{ki/wt}$*: 6.25%*

Taking into account the typical litter size of C57BL/6 mice of about 6-8 young animals, huge numbers of breeding pairs would be necessary to guarantee sufficient animal numbers (statistically, there would be less than one animal per litter with homozygously floxed Hif2a and Cre expression). A breeding scheme with such high numbers of surplus animals has not been included in our animal testing application as the German rules are very restrictive and to the best of our knowledge this experiment would not have been approved by the local authorities. We highly respect the rules of Russel and Burke to reduce the numbers of experimental animals. Taking into account that the viral loads of wildtype C57BL/6 animals infected with the same virus stocks as our Hif-1a^{fl} mice (please refer to the provided data in our first point-to-point response) we are deeply convinced that this would not justify breeding such high numbers of surplus animals.

3.- In this revised version of the manuscript, authors claim that Atp6v1e2 expression is not reduced upon HIF2a gene inactivation in Hif-2afl x LysM+/cre BMDMs. However, in Suppl. Figure 2, it seems that there is trend of reduced Atp6v1e2 expression (panel B) importantly in conditions in which HIF2 expression is partially reduced (panel A).

Authors should comment, why HIF2a expression is not completely reduced in Hif-2afl x LysM+/cre BMDMs?. Moreover, it is important to compare HIF2a expression in Hif- 2afl mice and control C57BL/6 without LoxP sites following the breeding scheme proposed in comment #2 above); or alternatively comparing Hif-1afl or Hif-2afl mice.

We thank the reviewer for raising these understandable concerns. We agree that there might be a trend towards a slightly reduced expression of Atp6v1e2 mRNA in Hif-2a^{fl} *x LysM$^{+/cre}$ BMDMs. This reduction is about 15% in suppl fig 2B. Nonetheless, this effect is far from reaching significance here (p=0.4667). We have added this explicitly to the respective figure legend now.*

The knockout efficiency in BMDMs shown in suppl figure 2A is indeed only about 40%, but comparable to the data we have published before for these mice (Kerber et al. 2020, doi: 10.3390/ijms21228551). In the cited publication, we increased the knockout efficiency by using mice with homozygous Cre expression. As the knockout efficiency is without relevance for the herein presented data, we have not used mice with homozygous Cre in this manuscript.

We fully agree with the reviewer that it is of great relevance to compare the expression of the Atp6v1e2 mRNA between Hif-1a^{fl} and Hif-2a^{fl} animals. We kindly refer to figure 5B in the manuscript for these data.

4.- The new title of the manuscript reflects now better the findings shown in this study, which suggest the role of vacuolar ATPase in macrophages in Friend virus infection. However, this conclusion is based on the use of Concanamycin A (Con A), which might be discussed as a limitation of the study.
This is an excellent point which we have added to our discussion as follows (ll. 392-395): "Our data are based on the use of ConA, an – although highly specific – inhibitor of the vacuolar ATPase and not e.g., on a direct knockdown of the *Atp6v1e2* gene, which has to be taken into account to prevent an overinterpretation of the herein presented findings. This is due to the fact that our initial focus was on the role of myeloid *Hif2a* in FV infection."

Third decision letter

MS ID#: JOCES/2023/261893

© 2024. Published by The Company of Biologists under the terms of the Creative Commons Attribution License (https://creativecommons.org/licenses/by/4.0/).

MS TITLE: Reduced vacuolar ATPase protects mice from Friend virus infection - an unintended but instructive effect in *Hif-2a*^{fl} mice

AUTHORS: Timm Schreiber, Nora Koll, Claudia Padberg, Buena Delos Reyes, Theresa Quinting, Anna Malyshkina, Eric Metzen, Kathrin Sutter, Joachim Fandrey, and Sandra Winning

I am happy to tell you that your manuscript has been accepted for publication in Journal of Cell Science, pending standard ethics checks.

© 2024. Published by The Company of Biologists under the terms of the Creative Commons Attribution License (https://creativecommons.org/licenses/by/4.0/).
